# IGF2 deficiency promotes liver aging through mitochondrial dysfunction and upregulated CEBPB signaling in d-galactose-induced aging mice

**DOI:** 10.1186/s10020-023-00752-0

**Published:** 2023-11-28

**Authors:** Xiaohai Zhou, Bowen Tan, Weiwei Gui, Caiping Zhou, Hanxin Zhao, Xihua Lin, Hong Li

**Affiliations:** Department of Endocrinology, the Affiliated Sir Run Run Shaw Hospital, School of Medicine, Zhejiang University, Hangzhou, China

**Keywords:** IGF2, Liver, Aging, Mitochondria dysfunction, CEBPB

## Abstract

**Background:**

Liver aging, marked by cellular senescence and low-grade inflammation, heightens susceptibility to chronic liver disease and worsens its prognosis. Insulin-like growth factor 2 (IGF2) has been implicated in numerous aging-related diseases. Nevertheless, its role and underlying molecular mechanisms in liver aging remain largely unexplored.

**Methods:**

The expression of IGF2 was examined in the liver of young (2–4 months), middle-aged (9–12 months), and old (24–26 months) C57BL/6 mice. In vivo, we used transgenic *IGF2*^*f/f*^*; Alb-Cre* mice and d-galactose-induced aging model to explore the role of IGF2 in liver aging. In vitro, we used specific short hairpin RNA against IGF2 to knock down IGF2 in AML12 cells. d-galactose and hydrogen peroxide treatment were used to induce AML12 cell senescence.

**Results:**

We observed a significant reduction of IGF2 levels in the livers of aged mice. Subsequently, we demonstrated that IGF2 deficiency promoted senescence phenotypes and senescence-associated secretory phenotypes (SASPs), both in vitro and in vivo aging models. Moreover, IGF2 deficiency impaired mitochondrial function, reducing mitochondrial respiratory capacity, mitochondrial membrane potential, and nicotinamide adenine dinucleotide (NAD)^+^/NADH ratio, increasing intracellular and mitochondrial reactive oxygen species levels, and disrupting mitochondrial membrane structure. Additionally, IGF2 deficiency markedly upregulated CCAAT/enhancer-binding protein beta (CEBPB). Notably, inhibiting CEBPB reversed the senescence phenotypes and reduced SASPs induced by IGF2 deficiency.

**Conclusions:**

In summary, our findings strongly suggest that IGF2 deficiency promotes liver aging through mitochondrial dysfunction and upregulated CEBPB signaling. These results provide compelling evidence for considering IGF2 as a potential target for interventions aimed at slowing down the process of liver aging.

**Supplementary Information:**

The online version contains supplementary material available at 10.1186/s10020-023-00752-0.

## Introduction

Aging is characterized by the gradual degeneration of physiological functions at the cellular, tissue, and organismal levels over time. This natural progression of aging is associated with increased susceptibility to age-related diseases and ultimately leads to an increased mortality risk (Calcinotto et al. 2019). In the liver, aging is characterized by morphological and physiological changes, including decreased volume, blood flow, and hepatic regenerative capacity (Schmucker 2005). As an important metabolic organ, the liver maintains whole-body homeostasis by regulating metabolic processes, clearing toxicants, and synthesizing molecules (Rui 2014). The changes in liver structure and function during the aging process increase the susceptibility to various types of chronic liver disease (CLD), including non-alcoholic fatty liver disease, non-alcoholic steatohepatitis, and hepatocellular carcinoma (HCC), and worsen their prognosis (Maeso-Diaz and Gracia-Sancho 2020). Currently, there is no approved pharmacological treatment for liver aging, emphasizing the need to understand its pathogenesis and identify new therapeutic targets.

Insulin-like growth factor 2 (IGF2) belongs to the IGF system and exhibits 70% homology with insulin-like growth factor 1 (IGF1) and nearly 50% amino acid sequence identity with insulin (Rotwein 1991). IGF2 is secreted mainly by the placenta during pregnancy, and after birth, its expression declines sharply, with most of this IGF2 thought to originate from the liver (Selenou et al. 2022; Vu and Hoffman 1994). An increasing body of evidence suggests that IGF2 plays a key role in cell proliferation and organ growth and participates in the development of numerous diseases, such as cancers, cardiovascular diseases, and CLD (Bergman et al. 2013; Adamek and Kasprzak 2018). Moreover, the past few years have witnessed a burgeoning interest in the role of IGF2 in age-related diseases. For instance, IGF2 or its analogs may be used to treat Huntington’s disease, Alzheimer’s disease, and age-related cognitive impairment (Beletskiy et al. 2021). Research conducted on rats to investigate the impact of IGF2 on cognitive decline associated with aging has provided evidence to support this notion. It was found that a single injection of IGF2 in the hippocampus significantly improved memory decay related to aging (Steinmetz et al. 2016). Furthermore, Muhammad et al. identified that aged mice exhibited reduced levels of IGF2 in the serum and oocytes, while IGF2 supplementation could increase mitochondrial functional activity and improve the developmental competency and meiotic structure of oocytes from aged mice (Muhammad et al. 2020). Another study found that IGF2 overexpression could accelerate the viability and depress the senescence of human dermal fibroblast cells (Tang et al. 2022). Although IGF2’s participation in the development of both CLD and age-related diseases has been established, little is known about the relationship between IGF2 and liver aging.

Mitochondria, known as the cellular powerhouses, play an important role in aging and age-related disease. In the liver, aging leads to a series of alterations in mitochondria. These alterations encompass mitochondrial DNA mutations, compromised oxidative phosphorylation (OXPHOS) capacity, increased oxidative stress and structural changes (Hunt et al. 2019). Meanwhile, mitochondrial dysfunction contributes to irreversible cell-cycle arrest and the development of senescence-associated secretory phenotypes (SASPs) (Miwa et al. 2022). SASPs comprise proinflammatory cytokines, chemokines, bioactive lipids, and damage-associated molecular patterns that can disrupt tissue microenvironments and ultimately lead to diverse aging-related pathologies and tissue dysfunction (Cai et al. 2022). Growing evidence has revealed the protective effects of IGF2 on maintaining normal mitochondrial function, preventing oxidative stress, and mitigating cellular damage (Castilla-Cortazar et al. 2011; Martin-Montanez et al. 2021). In line with these studies, our recent findings demonstrated that the knockdown of IGF2 in liver cells impairs mitochondrial function (Gui et al. 2021). These results imply that changes in IGF2 are likely to play a role in liver aging associated with mitochondrial function.

In this study, we aim to investigate the role of IGF2 in the liver aging. To achieve this, we assessed IGF2 levels in the livers of mice spanning different age groups, aiming to discern how its expression changes with advancing age. Furthermore, we employed in vitro and in vivo aging models to investigate the impact of inhibiting IGF2 on the aging process in the liver. This research could potentially serve as a valuable foundation for identifying novel targets for interventions aimed at retarding the progression of liver aging.

## Materials and methods

### Animal experiments

Animal care and experiments were conducted in accordance with the guidelines of the Animal Care Committee of Zhejiang University. Male C57BL/6 mice aged 8–10 weeks were purchased from the Model Animal Research Center of Nanjing University for the investigations. The mice were divided into three groups based on age: young group (2–4 months), middle-aged group (9–12 months), and old group (24–26 months). The livers were collected from mice of different ages.

To selectively inactivate the *IGF2* gene in the liver, CRISPR-Cas9 technology was used to insert loxP sites on flanking exons 2 of the *IGF2* gene in fertilized mouse eggs to generate *IGF2* floxed (*IGF2*^*f/f*^) mice (CKOCMS190327JN1 + CKOCMS190327JN2-B, Cyagen Biosciences). *IGF2*^*f/f*^ mice were crossbred with *Albumin-cre (Alb-cre)* mice (Strain #:003574, The Jackson Laboratory) to generate *IGF2*^*f/*+^*;Alb-Cre* (*IGF2*^*f/*+^*Cre*) mice. Then, *IGF2*^*f/*+^*Cre mice* were crossbred with *IGF2*^*f/f*^ mice to generate liver-specific *IGF2*-deficient mice, termed as *IGF2*^*f/f*^*; Alb-Cre* (*IGF2*^*f/f*^*Cre*). The *IGF2*^*f/f*^ littermates were used as controls (*IGF2*^*f/f*^). Genotyping for the *IGF2* floxed allele, and *Alb-Cre* gene was performed by PCR and genome sequencing using DNA extracted from tails. All mice used in the study were bred on a C57BL/6 background. Mice were maintained under standard conditions of 22 ± 2 °C, 50% to 60% relative humidity, and a 12-h light–dark diurnal cycle (lights on at 6: 00 A.M.), and had free access to water and food. High doses of d-galactose (d-gal) are widely used to establish mouse aging models and to explore the mechanisms underlying liver aging (Azman et al. 2021). 8-week-old male mice (25 ± 2 g) were randomly assigned into four groups (n = 5–7) according to genotype and whether they were administrated with d-gal: *IGF2*^*f/f*^, *IGF2*^*f/f*^*Cre*, *IGF2*^*f/f*^ + d-gal, and *IGF2*^*f/f*^*Cre* +  d-gal. The d-gal model group received intraperitoneal injections of 120 mg/kg/day of d-gal (59-23-4, Sigma-Aldrich) at a concentration of 12 g/L for 8 weeks, whereas the mice in the saline control group received equivalent volumes of physiological saline. Mice were sacrificed at the end of the experiments, and the livers of the mice were collected.

### Glucose and insulin tolerance tests

For the intraperitoneal glucose tolerance testing (IPGTT), mice were fasted for 16 h and then were given glucose (2 g/kg body weight). For the insulin tolerance test (ITT), mice received an intraperitoneal injection of insulin (1 IU/kg body weight) after 4 h of fasting. The blood glucose level from the tail vein was measured at 0 (baseline), 15, 30, 60, and 120 min; glucose levels were immediately measured using One Touch Ultra glucose strips (LifeScan).

### Enzyme-linked immunosorbent assay

The serum insulin levels were detected by double-antibody sandwich enzyme-linked immunosorbent assay (ELISA) using a wide range mouse insulin immunoassay kit (MS300, EZassay) following the manufacturer’s instructions.

### Assessment of liver function and histology

Plasma levels of alanine aminotransferase (ALT) and aspartate aminotransferase (AST) were measured using an Alanine aminotransferase Assay Kit (C009-2-1, Nanjing JianCheng) and an Aspartate aminotransferase Assay Kit (C010-2-1, Nanjing JianCheng) according to the manufacturer’s protocol, respectively. The liver tissue samples were preserved in 4% paraformaldehyde, followed by embedding in paraffin. Sections of 4 μm thickness were then obtained and subjected to hematoxylin and eosin (H&E) staining for histological analysis.

### RNA-Seq and analysis

Global transcriptome profiling was performed by RNA-Seq using liver tissues of *IGF2*^*f/f*^ +  d-gal and *IGF2*^*f/f*^*Cre* + d-gal mice (n = 3). RNA was extracted, sequenced, and analyzed by a custom service provided by Novogene using an Illumina NovaSeq 6000. Bioinformatics analysis was performed using the OmicStudio tools at https://www.omicstudio.cn/tool. The cut-off for differential expression threshold was based on |log2fold change (log2FC) |> 1 and *P*-value < 0.05.

### Cell culture

The immortalized normal mouse hepatocyte cell line (AML12) was purchased from the American Type Culture Collection and cultured in Dulbecco’s modified Eagle’s medium (DMEM)/F12 culture medium (GNM12500, Genomcell.bio), containing 10% fetal bovine serum (S-FBS-EU-015,SERANA), 100 U/mL Penicillin–Streptomycin (BL505A, Biosharp), 1% Insulin–Transferrin–Selenium (C0345, Beyotime), and 40 ng/mL dexamethasone (D4902, Sigma-Aldrich) at 37 ℃ in 5% CO_2_. For induction of cellular senescence, cells were exposed to 20 g/L d-gal for 48 h or exposed to 100 µM hydrogen peroxide (H_2_O_2_) for 2 h and cultured for 2 days in normal media. To elevate nicotinamide adenine dinucleotide (NAD^+^) levels, nicotinamide riboside (NR) (1341-23-7, Zhengzhou Acme Chemical Co. Ltd) at a dosage of 1 mM was supplemented to the culture medium for 48 h before measurement.

### Cells infection and transfection

For infection and transfection, AML12 cells were seeded in 6-well plates (10^5^ cells/mL) and cultured overnight (30–40% confluency). The specific short hairpin RNA (shRNA) against IGF2 (sh-IGF2) and its corresponding negative control (sh-NC), as well as their lentiviral vector construction, were acquired from Genechem (Shanghai, China). Cells were infected with lentivirus with a multiplicity of infection (MOI) of 10 and incubated with 6 μg/mL polybrene for 12 h to increases the efficiency of infection. Then, the supernatant was replaced by the growth medium. When the cells reached 80% confluency, 3 μg/mL puromycin was added to the culture medium to eliminate uninfected cells. When stably transfected cells appeared, the concentration of puromycin in the culture medium was reduced to 1 ug/mL for continuous screening of infected cells. To knock down CEBPB, small interfering RNAs (siRNAs) were used. Cells were transiently transfected with si-CEBPB or si-NC (TSINGKE, Beijing, China) via Lipo 8000 (C0533, Beyotime) according to the manufacturer’s protocol. Cells were harvested after transfection for 48 h. The sequences of shRNA and siRNA are listed in Additional file 1: Table S1.

### RNA isolation and real-time quantitative PCR

Total RNA was isolated from AML12 cells and mouse liver tissues using AG RNAex Pro reagent (AG21102, Accurate Biotechnology) and reverse transcribed using an Evo M-MLV RT Premix kit (AG11706, Accurate Biotechnology) according to the manufacturer’s protocols. Real-time quantitative PCR (RT-qPCR) was performed utilizing the SYBR Green Premix Pro Taq HS qPCR kit (AG11701, Accurate Biotechnology) on a LightCycler 480 PCR System (Roche, Basel, Switzerland). The relative mRNA expression levels were calculated using the 2^−ΔΔCT^ method and normalized to GAPDH mRNA as the internal control. The various primer sets used in this study are shown in Additional file 1: Table S2.

### Western blotting

Total protein was extracted from AML12 cells and mouse liver tissues using RIPA lysis (FD009, FdBio Science) containing protease and phosphatase inhibitors, and protein concentrations were measured via BCA Protein Assay Reagent (FD2001, FdBio Science). Equal amounts of proteins were denatured and subjected to 10% or 12% sodium dodecylsulfate-polyacrylamide gel electrophoresis (SDS-PAGE), transferred to a polyvinylidene difluoride membrane, and blocked with 5% non-fat milk for 1 h at room temperature. After blocking, the membranes were incubated overnight with primary antibodies at 4 °C. Primary antibodies were diluted at 1:1000 and included IGF2 (ab9574, Abcam), P53 (10442-1-AP, Proteintech), P21 (A11454, Abclonal), P16 (A0262, Abclonal), CEBPB (sc-7962, Santa Cruz Biotechnology), AKT (9272S, Cell Signaling Technology), p-AKT (4060S, Cell Signaling Technology), ERK1/2 (4695S, Cell Signaling Technology), p-ERK1/2 (4370S, Cell Signaling Technology), p38 (8690S, Cell Signaling Technology), p-p38 (4511S, Cell Signaling Technology). After washing, the membranes were incubated with a secondary anti-mouse (FDM007, FdBio Science; diluted at 1:5000) or anti-rabbit antibody (FDR007, FdBio Science; diluted at 1:5000) for 1 h at room temperature, and protein signals were visualized using an enhanced chemiluminescence kit (FD8030, FdBio Science).

### Senescence-associated-β-galactosidase assay

The senescence-associated-β-galactosidase (SA-β-gal) assay was performed using a SA-β-gal Staining Kit (C0602, Beyotime) according to the manufacturer’s instructions. In brief, frozen liver sections and AML12 cells were prepared, washed, fixed, and stained with the β-galactosidase staining solution in a 37 °C dry incubator (without CO_2_). The SA-β-gal-positive cells exhibited a blue color, and photos were taken with an optical microscope (Olympus, Tokyo, Japan).

### Immunofluorescence

After de-waxing and rehydration, liver sections were antigen retrieved in Tris/EDTA buffer (pH 9.0) for 20 min. AML12 cells seeded on coverslips were fixed in 4% paraformaldehyde for 20 min, permeabilized with 0.2% Triton X-100 for 15 min. After blocking the liver sections and cells with 5% bovine serum albumin (FD0030, FdBio Science) for 1 h, they were incubated with primary antibodies against F4/80 (13-4801-82, Invitrogen; diluted at 1:100) and γ-H2AX (bs-2560R, Bioss; diluted at 1:200) overnight at 4 °C. After removing the primary antibody by washing, they were incubated with DyLight 488 AffiniPure Goat Anti-Mouse IgG (FD0150, FdBio Science; diluted at 1:500) or DyLight 594 AffiniPure Goat Anti-Rabbit IgG (FD0129, FdBio Science; diluted at 1:500) for 1 h at room temperature, and counterstained with 4′,6-diamidino-2-phenylindole (DAPI) for 5 min. Observation and photo-taking were conducted using a fluorescence microscope (Olympus, Tokyo, Japan), and fluorescence intensity was measured using Image-J software.

### Cell viability assay

Cell viability was determined by the cell counting kit-8 assay (CCK-8; C0038, Beyotime). AML12 cells were seeded at a density of 0.5 × 10^4^ cells per well in 96-well plates. After cell treatment, the culture supernatant was removed, and the cells were incubated in a culture medium containing CCK8 for 2 h at 37 °C in the dark. The absorbance at 450 nm was measured by a Multiskan GO microplate reader (Thermo Fisher Scientific, Waltham, MA, USA).

### Cell cycle analysis

The cell cycle and apoptosis were analyzed using commercially available kits (C1052, Beyotime) according to the manufacturer’s instructions. Briefly, AML12 cells were harvested, fixed with 70% ethanol overnight at 4 °C, and incubated with propidium iodide staining solution in the dark for 30 min. The cell cycle analysis was performed using a flow cytometer (BD Biosciences, San Jose, CA, USA), and the data were analyzed using ModFit LT 4.1 software.

### Measurement of oxygen consumption rate

Oxygen consumption rates of AML12 cells were examined using the XF Mito Stress Test Kit (103015-100, Agilent) on an XFe96 Extracellular Flux Analyzer following the manufacturer’s protocols. One day before the assay, 8000 cells per well were seeded in XF 96-wells microplates, and a sensor cartridge was hydrated in XF Calibrant at 37 °C in a CO_2_-free incubator overnight. On the day of the assay, the cells were incubated in 180 mL of XF assay medium (XF Base Medium supplemented with 1 mM pyruvate, 2 mM l-glutamine, and 10 mM glucose adjusted to pH 7.4) at 37 °C in a non-CO_2_ incubator for 1 h before initiation of measurements. During the incubation period, 10 × stocks of drugs in XF media were successively loaded into the injection ports in the XFe 96 sensor cartridge, and the final concentrations of drugs after injections were: 1.0 μM Oligomycin, 1.0 μM Carbonyl cyanide 4-(trifluoromethoxy)phenylhydrazone (FCCP), and 0.5 μM Rotenone/antimycin A (Rot/AA). The plates were assayed using an XFe96 analyzer to measure the oxygen consumption rate (OCR) over time. This was done by sequentially adding different drugs and monitoring the OCR response. The OCR of cells was normalized to cell number, and datasets were analyzed by Wave software (Agilent).

### Measurement of ROS production

Reactive oxygen species (ROS) production was identified using a ROS assay kit (S0033S, Beyotime) according to the manufacturer’s instructions. Briefly, AML12 cells were washed with PBS and incubated with 10 μM 2ʹ,7ʹ-Dichlorodihydrofluorescein diacetate (DCFH-DA) probes without light for 30 min at 37 °C. The cells were washed twice with PBS, resuspended in PBS, and subjected to flow cytometric analysis (BD Biosciences, San Jose, CA, USA).

### Measurement of mitochondrial membrane potential

Mitochondrial membrane potential (MMP) was detected using the JC-1 assay kit (M8650, Solarbio) following the manufacturer’s instructions. AML12 cells were stained live in a growth medium with the fluorescent probe JC-1 (1:200) at 37 °C for 20 min. After washing, the cells were resuspended in PBS and subjected to flow cytometric analysis (BD Biosciences, San Jose, CA, USA).

### Mitochondrial ROS

The mitochondrial ROS was detected using the MitoSOX™ Red mitochondrial superoxide indicator (M36008, Invitrogen). AML12 cells cultured in 12-well plates were incubated with 5 μM MitoSOX™ reagent working solution for 10 min at 37 °C, protected from light. Cells were washed with PBS three times, and the images were captured using an fluorescence microscope (ZEISS, Oberkochen, Germany).

### Transmission electron microscopy

AML12 cells were fixed with 2.5% glutaraldehyde in PBS buffer for 2 h at room temperature, followed by overnight fixation at 4 °C. The cells were then post-fixed in 1% osmic acid at 37 °C for 1 h and stained with aqueous 2% uranyl acetate for 30 min. After dehydration with an ascending gradual series of ethanol (50%, 70%, 90%, and 100%) for 15 min each, the samples were transferred to absolute acetone for 20 min. Cells were further penetrated with a 1:1 mixture of absolute acetone and embedding agent for 2 h and then embedded in a pure embedding agent. The samples were cut into approximately 100-nm-thick ultrathin sections with a Leica UC7 ultramicrotome and examined with a transmission electron microscope (Tecnai T10, Holland).

### ***Measurement of NAD***^+^***/NADH ratios***

NAD^+^ and NADH were quantified using a commercial kit (N6035, UElandy). 1 × 10^6^ cells were treated with 200 μL of NAD^+^ /NADH extraction buffer. To detect the total amounts of NAD^+^ and NADH, 20 μL of extracted samples were transferred into 96-well plates. To detect NADH, 20 μL of extracted samples were heated to 60 °C for 30 min and transferred into 96-well plates. Then, the samples were mixed with enzyme mix, NADH developer and NAD buffer, and OD450 was measured. Standard curves (0–200 pmol) were generated for quantification.

### Statistical analysis

All experiments were repeated in triplicate. Statistical analysis was performed using GraphPad Prism 8 software. Continuous data were expressed as means ± SEM. Comparisons between the two groups were made using unpaired student t-tests. Differences between more than two independent groups were analyzed using one-way ANOVA. A *P*-value < 0.05 was statistically significant.

## Results

### Liver tissues of old mice exhibited downregulated IGF2 expression, but upregulated senescent genes and SASPs expression

**Fig. 1:**
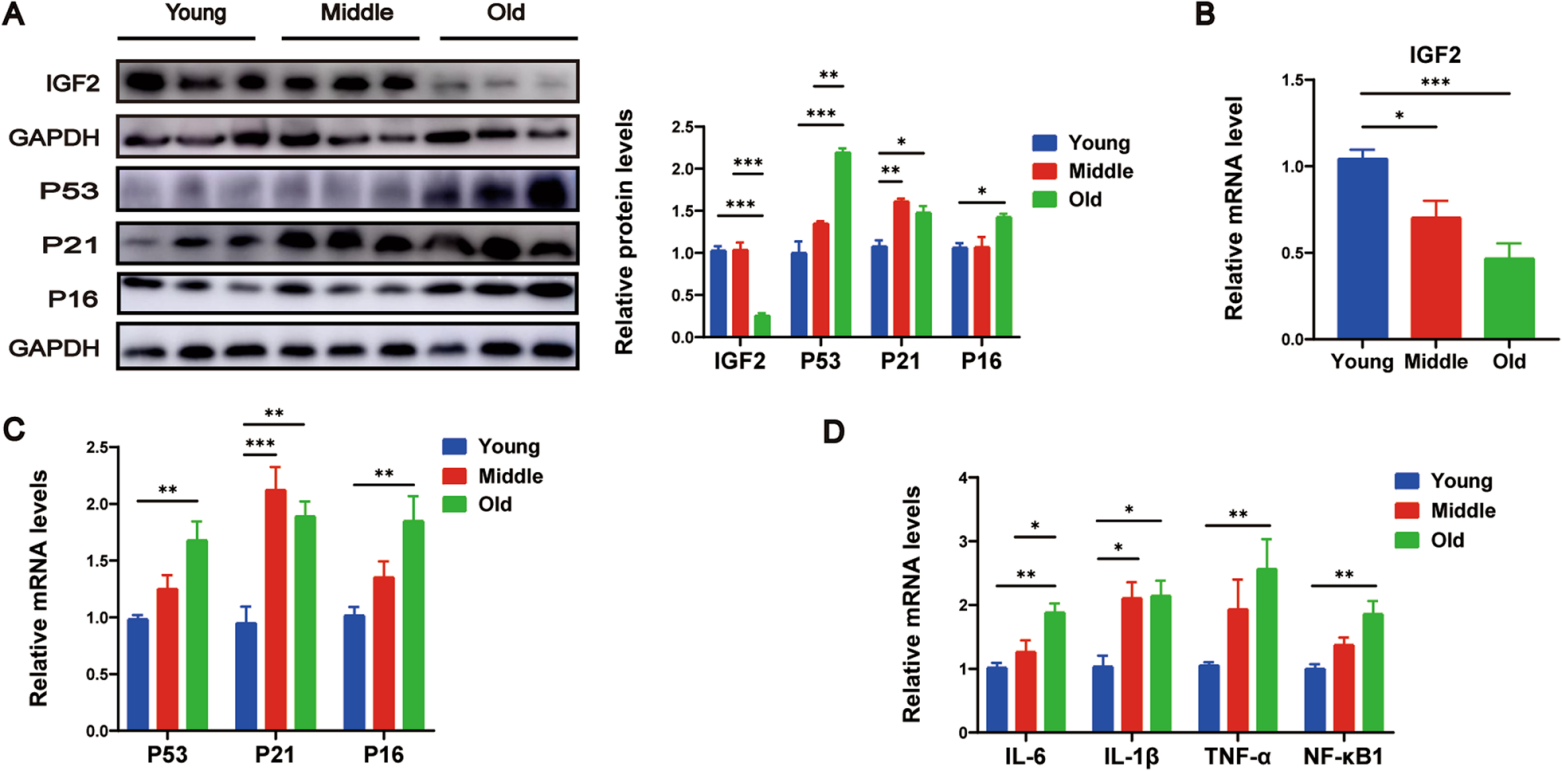
Liver tissues of old mice exhibit decreased expression of IGF2 and aging liver microenvironment. **A** Western blotting analysis for the protein expression of IGF2 and key senescence-associated genes P53, P21 and P16 in the liver from mice of different ages. The quantitative data are presented. mRNA levels of IGF2 (**B**), senescence-associated genes P53, P21 and P16 (**C**), and senescence-associated secretory phenotype genes IL-6, IL-1β, TNF-α and NF-κB1 (**D**), as determined by RT‒qPCR analysis. n = 5 per group. Data are shown as means ± SEM. One-way ANOVA was used for comparison among multiple groups. ^*^P < 0.05, ^**^P < 0.01, ^***^P < 0.001

Initially, we harvested liver samples from mice of different ages, including young (2–4 months), middle-aged (9–12 months), and old (24–26 months) mice, to detect IGF2 expression. Western blot analysis revealed that the old mice had significantly reduced IGF2 protein levels (Fig. 1A). RT‒qPCR analysis further confirmed the decreased expression of IGF2 mRNA in the old mice (Fig. 1B).

To confirm the presence of an aging liver microenvironment, we examined the protein and mRNA levels of key senescence-associated genes, namely P53, P21 and P16. Our results demonstrated elevated expression levels of these genes in the old mice (Fig. 1A and C. Furthermore, we evaluated the expression of various SASPs and observed elevated levels of IL-6, IL-1β, TNF-α, and NF-κB1 in the old mice (Fig. 1D). These findings unequivocally demonstrate that the liver exhibits senescent characteristics with age.

### Conditional knockout of IGF2 in hepatocyte accelerated d-gal-induced liver aging

To determine the role of IGF2 in the pathogenesis of liver aging, we generated conditional knockout mice, as described in the methods section.

**Fig. 2:**
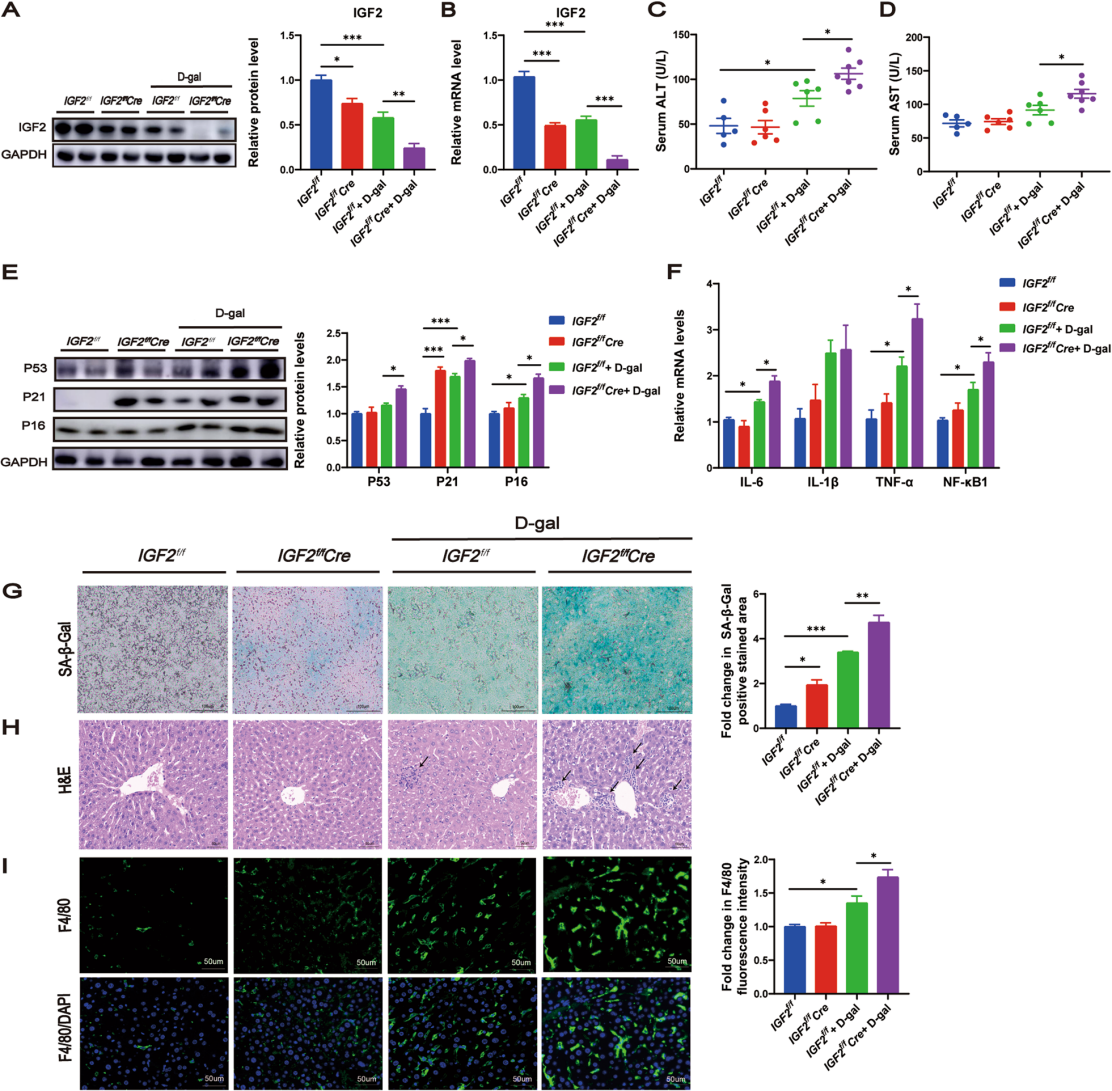
Conditional knockout of IGF2 in hepatocyte accelerated d-gal-induced liver aging. The expression levels of IGF2 were examined by Western blotting (**A**) and RT‒qPCR (**B**). Liver function evaluated by ALT (**C**) and AST (**D**). **E** Western blotting analysis for the protein level of senescence-associated genes P53, P21 and P16 in the liver tissues. The quantitative data are presented. **F** Relative mRNA expression levels of senescence-associated secretory phenotype genes IL-6, IL-1β, TNF-α and NF-κB1 in the liver tissues, as measured by RT‒qPCR. **G** SA-β-gal staining of liver sections. β-gal staining area analysis was shown. Scale bar, 100 um. **H** H&E staining of liver sections. Scale bar, 50 um. **I** Immunofluorescent staining of F4/80 and DAPI on liver sections. Fluorescence intensity analysis was shown. Scale bar, 50 um. **A**–**F** n = 5–7 per group, **G**–**I** n = 3 per group. All values are shown as means ± SEM. Dots represent individual level data. One-way ANOVA was used for comparison among multiple groups. ^*^P < 0.05, ^**^P < 0.01, ^***^P < 0.001

CRISPR-Cas9 technology was used to insert loxP sites on flanking exons 2 of the *IGF2* gene and recombination with *Alb-Cre* to generate conditional knockout of IGF2 in hepatocytes (Additional file 2: Figure S1A). PCR analysis identified different genotypes, including *IGF2*^+*/*+^ (one band with 204 bp), I*GF2*^*f/*+^ (two bands with 250 bp and 204 bp), *IGF2*^*f/f*^ (one band with 250 bp), and with *Alb-Cre* negative (no band) or *Alb-Cre* positive (one band with 390 bp) (Additional file 2: Figure S1B). Genome sequencing also confirmed that loxP sites were successfully inserted in *IGF2*^*f/f*^ (Additional file 2: Figure S1C). Western blotting and RT‒qPCR analyses were conducted to evaluate knockout efficiency. We found that lower IGF2 levels were present in the *IGF2*^*f/f*^*Cre* mice than in the *IGF2*^*f/f*^ mice (Fig. 2A, B). Importantly, d-gal-treated mice displayed lower IGF2 levels than saline-treated mice, and *IGF2*^*f/f*^*Cre* + d-gal mice had the lowest IGF2 levels (Fig. 2A, B).

Lower body weight change (Additional file 2: Figure S1D), body weight gain (Additional file 2: Figure S1E), and ratios of liver/body weight (Additional file 2: Figure S1F) were observed in *IGF2*^*f/f*^*Cre* mice compared with *IGF2*^*f/f*^ mice both in the saline control group and d-gal model group. Glucose tolerance (Additional file 2: Figure S1G, H), fasting glucose (Additional file 2: Figure S1K), and fasting insulin (Additional file 2: Figure S1L) in the *IGF2*^*f/f*^ and *IGF2*^*f/f*^*Cre* mice were comparable between the saline control group and d-gal model group, whereas the *IGF2*^*f/f*^*Cre* mice showed better insulin sensitivity compared to *IGF2*^*f/f*^ mice in the d-gal model group (Additional file 2: Figure S1I-J). d-gal serves as a reliable aging model for inducing liver aging, evidenced by increased senescence markers, inflammation, and liver damage (Azman et al. 2021). To assess liver damage, we detected serum levels of transaminases. We found that d-gal model group mice displayed elevated ALT levels compared to saline control group mice (Fig. 2C). ALT and AST levels of *IGF2*^*f/f*^ and *IGF2*^*f/f*^*Cre* were similar in the saline control group, but their levels of *IGF2*^*f/f*^*Cre* mice were increased compared to those of *IGF2*^*f/f*^ mice in the d-gal model group (Fig. 2C, D). Furthermore, We examined the expression levels of crucial senescence-associated genes and observed that d-gal treatment elevated P21 and P16 expression (Fig. 2E). Moreover, knockout of IGF2 resulted in elevated P21 expression in the saline control group, and led to increased expression of P53, P21 and P16 in the d-gal model group (Fig. 2E). In addition, our findings revealed that d-gal treatment elevated the expression of several SASPs, such as IL-6, TNF-α and NF-κB1, and knockout of IGF2 further increased the expression of these SASPs in the d-gal model group (Fig. 2F). SA-β-gal is defined as beta-galactosidase activity detectable at pH 6.0 in senescent cells, which is currently a widely used biomarker of senescence (Lee et al. 2006). Staining for SA-β-gal of the frozen liver sections showed that d-gal treatment enhanced SA-β-gal activity, and IGF2 knockout enhanced SA-β-gal activity both in the saline control group and the d-gal model group (Fig. 2G). H&E staining showed that d-gal treatment promoted hepatocytes swelling and inflammatory cells infiltration, and *IGF2*^*f/f*^*Cre* mice showed more inflammatory infiltration compared to *IGF2*^*f/f*^ mice in the d-gal model group (Fig. 2H). Immunofluorescent staining of F4/80 confirmed that d-gal treatment elevated macrophage infiltration, and IGF2 knockout further elevated macrophage infiltration and accelerated the development of an inflammatory microenvironment in the d-gal model group (Fig. 2I).

Collectively, these findings provide compelling evidence that the absence of IGF2 in the liver not only exacerbates d-gal-induced liver aging but also stimulates the production of SASPs.

### IGF2 deficiency enhanced the transcription of senescence and inflammation genes but impaired the transcription of mitochondrial function genes

**Fig. 3:**
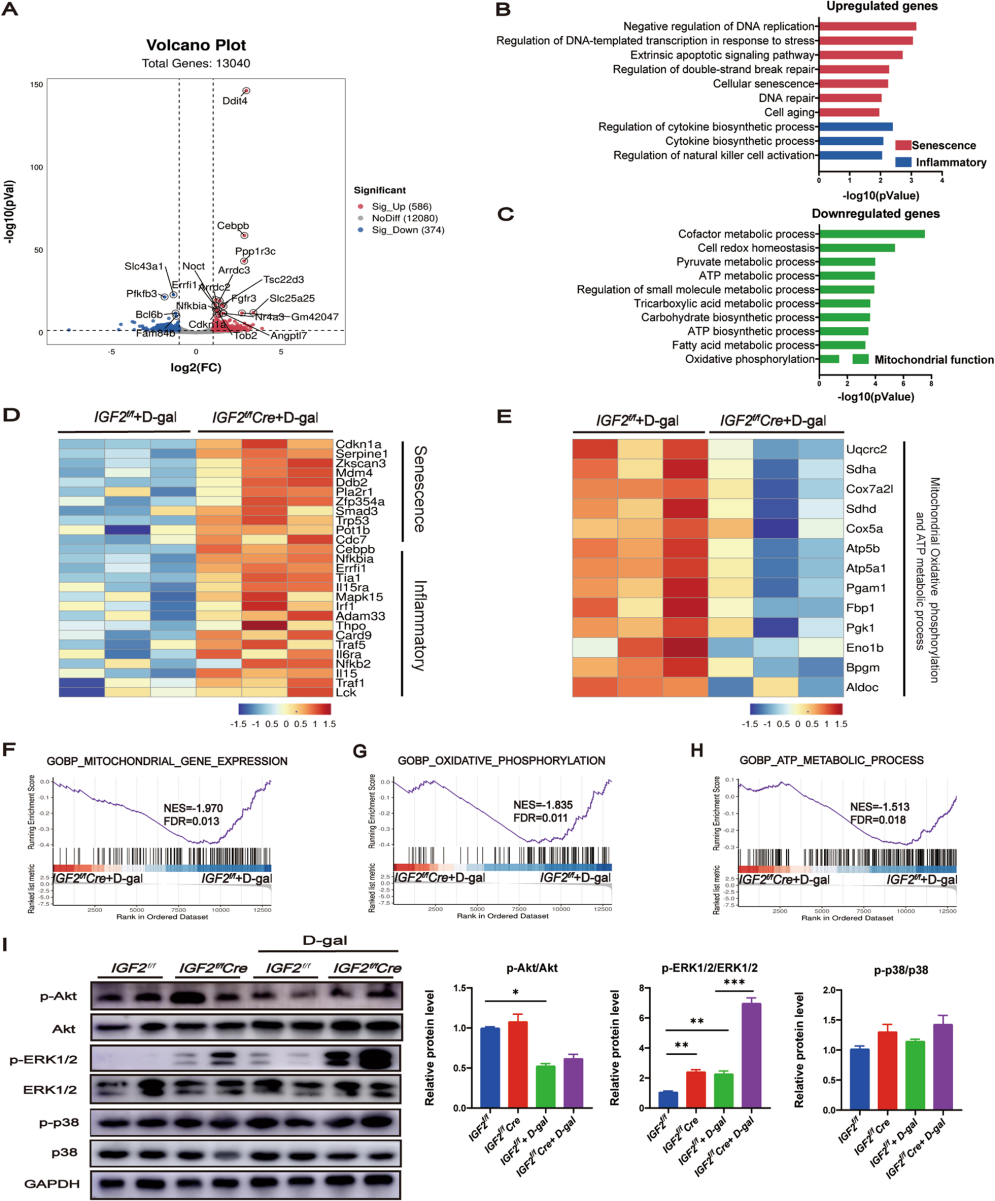
IGF2 deficiency regulated the transcription of senescence, inflammation and mitochondrial function. RNA-Seq was performed in the liver tissues of *IGF2*^*f/f*^ + d-gal and *IGF2*^*f/f*^*Cre* + d-gal mice (n = 3 per group). **A** Volcano plot showing DEGs and labeling top 20 DEGs. The red and blue dots indicated upregulated and downregulated genes, respectively. GO analysis of the upregulated DEGs (**B**) and downregulated DEGs (**C**). A heatmap showed genes associated senescence and inflammatory (**D**), mitochondrial oxidative phosphorylation and ATP metabolic process (**E**). GSEA and pathway enrichment analysis of pathways related to mitochondrial gene expression (**F**), oxidative phosphorylation (**G**), and ATP metabolic process (**H**). **I** Western blotting analysis for the protein levels of AKT, p-AKT, ERK1/2, p-ERK1/2, p38 and p-p38 (n = 5–7 per group). The quantitative data are presented. Data are shown as means ± SEM. One-way ANOVA was used for comparison among multiple groups. ^*^P < 0.05, ^**^P < 0.01, ^***^P < 0.001

To further elucidate the role of IGF2 deficiency in liver aging, we analyzed the transcriptomic changes of the liver tissues of *IGF2*^*f/f*^ + d-gal and *IGF2*^*f/f*^*Cre* + d-gal mice. Our in silico analysis uncovered 960 differentially expressed genes (DEGs), including 586 upregulated and 374 downregulated DEGs (Fig. 3A). Gene Ontology (GO) analysis revealed that the upregulated genes mainly regulated pathways related to senescence and inflammation, such as negative regulation of DNA replication, extrinsic apoptotic signaling pathway, cellular senescence, and regulation of cytokine biosynthetic process (Fig. 3B). On the other hand, the downregulated genes mainly regulated the pathways related to mitochondrial function and mitochondria-related metabolism, such as the cofactor metabolic process, pyruvate metabolic process, ATP metabolic process, carbohydrate biosynthetic process, ATP biosynthetic process, fatty acid metabolic process and oxidative phosphorylation (Fig. 3C). Kyoto Encyclopedia of Genes and Genomes (KEGG) analysis (Additional file 3: Figure S2A, B) and heatmap analysis (Fig. 3D, E) revealed comparable findings, with upregulated DEGs linked to senescence and inflammation, and downregulated DEGs related to mitochondrial functions. Gene set enrichment analysis (GSEA) and pathway enrichment analysis showed that mitochondrial gene expression, oxidative phosphorylation, and ATP metabolic process pathways were significantly downregulated in *IGF2*^*f/f*^*Cre* + d-gal mice (Fig. 3F–H). IGFs primarily exert their effects by interacting with the IGF1R receptor (Chao and D'Amore 2008). In our study, we investigated the primary downstream signaling pathways of IGF1R and observed that d-gal diminished Akt signaling but upregulated ERK1/2 signaling (Fig. 3I). Importantly, IGF2 knockout in hepatocytes did not significantly affect AKT and p38 signaling but significantly upregulated the ERK1/2 signaling, independently of d-gal treatment (Fig. 3I).

### IGF2 knockdown stimulated senescence and senescence-associated secretory phenotypes in vitro

**Fig. 4:**
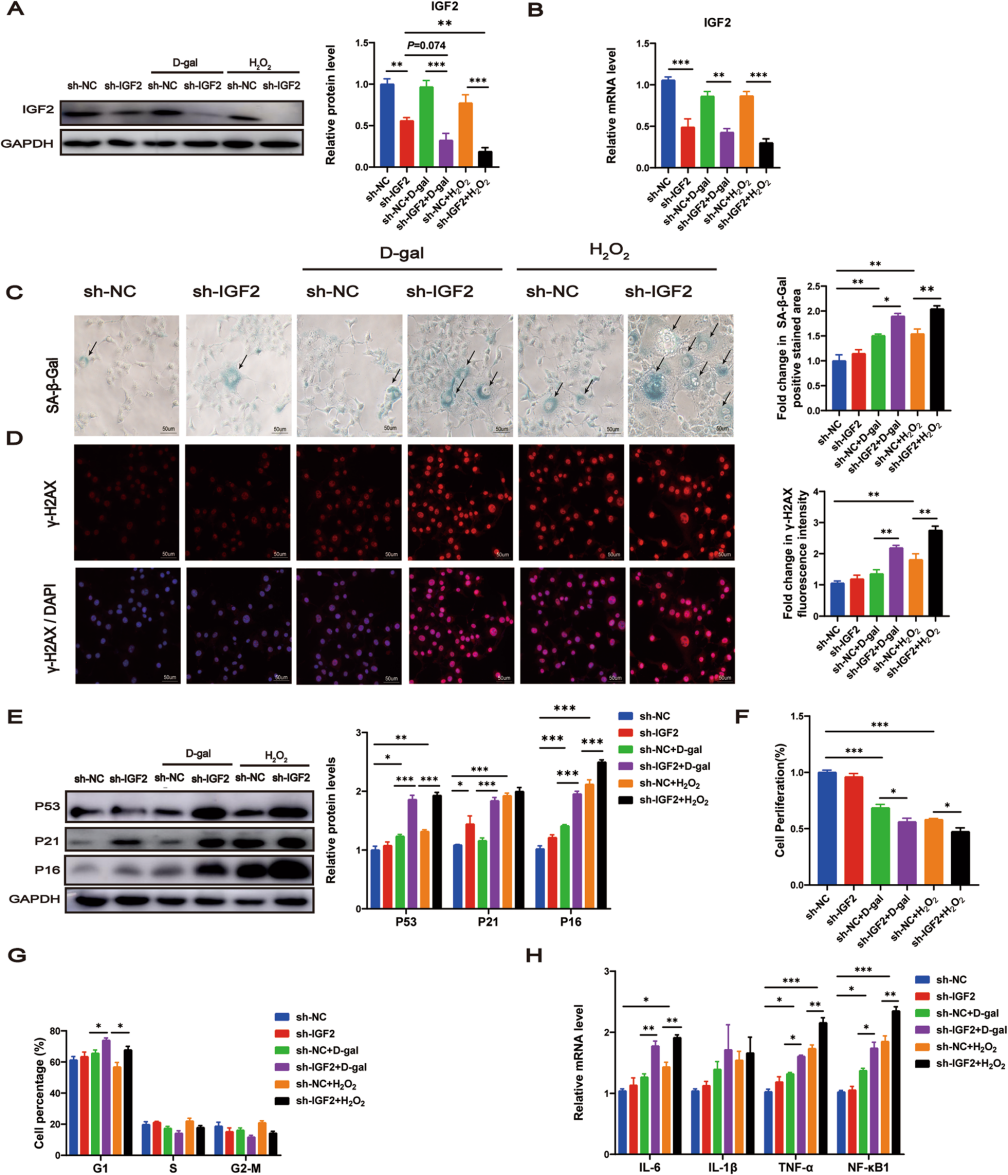
IGF2 knockdown stimulates senescence and senescence-associated secretory phenotypes in d-gal and H_2_O_2_ treated AML12 cells. AML12 cells were transfected with sh-IGF2 or its corresponding negative control sh-NC treated with d-gal, H_2_O_2_ or the PBS control. Western blotting analysis (**A**) and RT‒qPCR analysis (**B**) for the expression of IGF2 protein and mRNA. **C** The senescence of AML12 cells detected by SA-β-gal staining. SA-β-gal staining area analysis was shown. Scale bar, 50 μm. **D** Immunofluorescence analysis of γ-H2AX expression in AML12 cells. Fluorescence intensity analysis was shown. Scale bar, 50 μm. **E** Western blot analysis to measure the senescence-associated genes P53, P21 and P16 in AML12 cells. The quantitative data are presented. **F** Cell viability of AML12 cells tested by CCK-8 assay. **G** The cell cycle distribution of AML12 cells analyzed by flow cytometry. **H** RT‒qPCR analysis of mRNA levels of senescence-associated secretory phenotype genes IL-6, IL-1β, TNF-α and NF-κB1. Data are shown as means ± SEM. One-way ANOVA was used for comparison among multiple groups. ^*^P < 0.05, ^**^P < 0.01, ^***^P < 0.001. All experiments were repeated three times independently

To further evaluate the effect of IGF2 on cellular senescence, AML12 cells were transfected with sh-IGF2 or its corresponding negative control sh-NC to knock down IGF2. d-gal or H_2_O_2_ was used to induce senescence in AML12 cells in vitro. Western blotting and RT‒qPCR revealed successful inhibition of IGF2 in AML12 cells (Fig. 4A, B). In addition, d-gal or H_2_O_2_ treatment further decreased IGF2 protein levels after AML12 cells transfected with sh-IGF2 (Fig. 4A).

Senescence-related phenotypes were detected to investigate whether IGF2 knockdown could promote AML12 cell senescence. SA-β-gal staining showed that IGF2 knockdown increased the SA-β-gal staining area in AML12 cells treated with d-gal or H_2_O_2_ (Fig. 4C). Immunofluorescence staining showed that IGF2 knockdown increased the markers of DNA damage response γ-H2AX in AML12 cells treated with d-gal or H_2_O_2_ (Fig. 4D). Moreover, IGF2 knockdown upregulated the expression of senescence-associated genes P53, P21 and P16 in AML12 cells treated with d-gal, and upregulated P53 and P16 in AML12 cells treated with H_2_O_2_ (Fig. 4E). Consistently, IGF2 knockdown reduced cell proliferation after senescence induction (Fig. 4F). As expected, IGF2 knockdown resulted in more cells in the G1 phase after senescence induction, suggesting that IGF2 knockdown blocks the cell cycle (Fig. 4G). In addition, when IGF2 was knocked down, we observed an upregulation of SASPs, including IL-6, TNF-α and NF-κB1, following the induction of senescence (Fig. 4H). Taken together, our findings suggest that IGF2 depletion promoted senescence and SASPs of AML12 cells in different senescence-inducing conditions.

### IGF2 knockdown in AML12 cells impaired mitochondrial functions

**Fig. 5:**
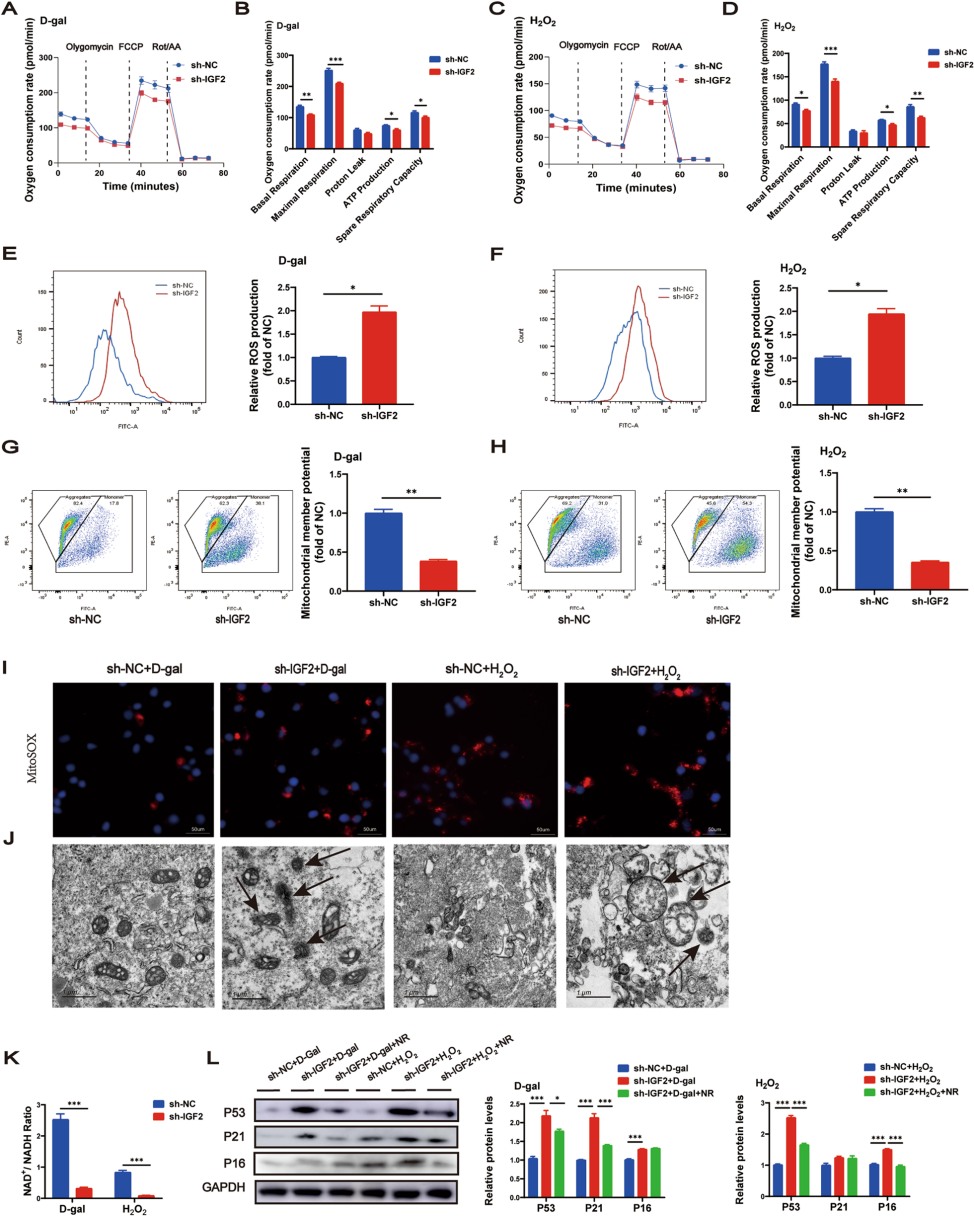
IGF2 knockdown in AML12 cells impairs mitochondrial functions. AML12 cells were transfected with sh-IGF2 or its corresponding negative control sh-NC treated with d-gal or H_2_O_2_. Representative oxygen consumption rates in AML12 cells treated with d-gal (**A**) or H_2_O_2_ (**C**). Basal and maximal respiration, proton leakage, ATP production and spare respiratory capacity in AML12 cells treated with d-gal (**B**) or H_2_O_2_ (**D**). Representative images for ROS production assessed via flow cytometric analyses, and the quantified ROS production are presented in AML12 cells treated with d-gal (**E**) or H_2_O_2_ (**F**). MMP was tested by flow cytometer, and the quantified MMP are presented in AML12 cells treated with d-gal (**G**) or H_2_O_2_ (**H**). **I** Mitochondrial ROS assessed by MitoSOX staining in AML12 cells treated with d-gal or H_2_O_2_. **J** Representative electron micrographs in AML12 cells treated with d-gal or H_2_O_2_. **K** The ratio of NAD^+^/NADH in AML12 cells treated with d-gal or H_2_O_2_. **L** Western blotting analysis for protein levels of P53, P21 and P16. The quantitative data are presented. Data are shown as means ± SEM. Student t tests was used for comparison between two groups. One-way ANOVA was used for comparison among multiple groups. ^*^P < 0.05, ^**^P < 0.01, ^***^P < 0.001. All experiments were repeated three times independently

To clarify the effect of IGF2 on mitochondrial function, AML12 cells were transfected with sh-IGF2 or its corresponding negative control sh-NC to knock down IGF2. Next, we used d-gal or H_2_O_2_ treatment to induce AML12 cell senescence in vitro. Seahorse analysis revealed that IGF2 knockdown inhibited basal and maximal mitochondrial respiration, ATP production, and spare respiratory capacity (Fig. 5A–D). Besides, IGF2 knockdown caused a significant increase in the production of intracellular ROS (Fig. 5E, F) and disrupted MMP (Fig. 5G, H). Importantly, IGF2 knockdown increased mitochondrial ROS (Fig. 5I). Furthermore, IGF2 knockdown resulted in abnormal mitochondria characterized by disrupted mitochondrial membrane structure (Fig. 5J). In addition, it has been reported that lower NAD^+^ levels and NAD^+^/NADH ratios drive mitochondrial dysfunction-associated senescence (Wiley et al. 2016). The present study found that IGF2 knockdown decreased NAD^+^/NADH ratios (Fig. 5K). To further clarify whether lower NAD^+^/NADH ratios caused the pro-aging effect of IGF2 knockdown, we performed NAD^+^ replenishment via supplementing with NR, an NAD^+^ precursor, to increase NAD^+^ content in AML12 cells treated with d-gal or H_2_O_2_. We observed that IGF2 knockdown upregulated the expression of senescence-related genes P53, P21 and P16 in AML12 cells exposed to d-gal, whereas NR supplementation decreased P53 and P21 expression, partially rescued pro-aging effect of IGF2 deficiency (Fig. 5L). Similarly, IGF2 knockdown elevated P53 and P16 expression in AML12 cells exposed to H_2_O_2_, but NR supplementation decreased P53 and P16 expression, rescued pro-aging effect of IGF2 deficiency (Fig. 5L).

In summary, our findings indicate that IGF2 deficiency leads to impaired mitochondrial respiratory function, increased intracellular and mitochondrial reactive oxygen species generation, reduced MMP and NAD^+^/NADH ratios, and impaired mitochondrial membrane structure. These mitochondrial dysfunctions contribute to the development of senescence and the secretion of SASPs. Importantly, NR replenishment rescued the senescence phenotypes induced by IGF2 deficiency to a certain extent.

### Upregulation of the CEBPB signaling was critical for the pro-aging effects of IGF2 deficiency in AML12 cells after senescence induction

**Fig. 6:**
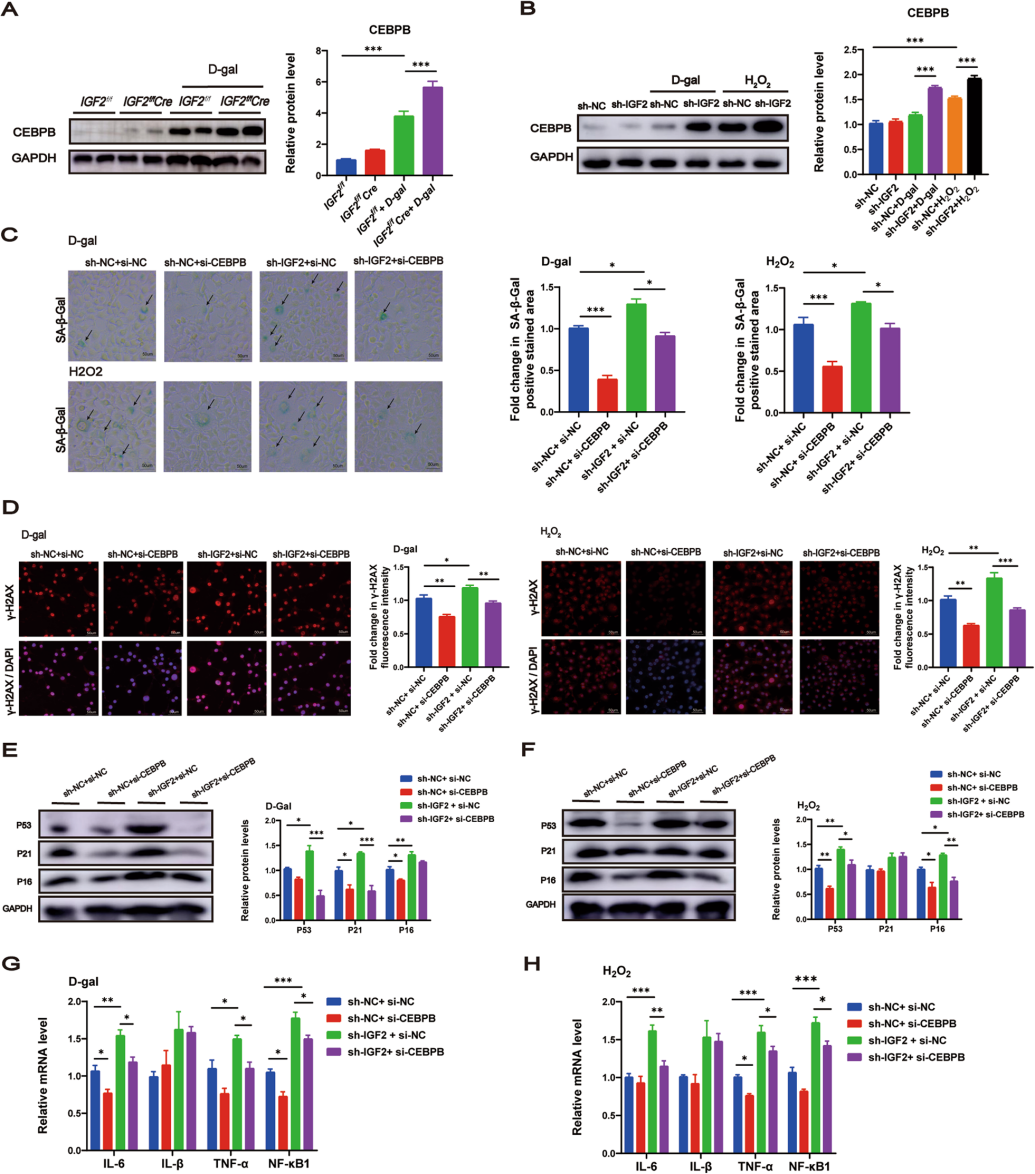
Up-regulation of the CEBPB signaling is critical for the pro-aging effects of IGF2 deficiency. **A** Western blot analysis of protein levels of CEBPB in *IGF2*^*f/f*^ and *IGF2*^*f/f*^Cre mice of saline control group and d-gal model group. The quantitative data are presented. n = 5–7 per group. **B** Western blot analysis of protein levels of CEBPB in AML12 cells transfected with sh-IGF2 or sh-NC and treated with d-gal, H_2_O_2_ or the PBS control. **C**–**H** AML12 cells were transfected with sh-NC + si-NC, sh-NC + si-CEBPB, sh-IGF2 + si-NC and sh-IGF2 + si-CEBPB, and treated with d-gal or H_2_O_2_. **C** The senescence of AML12 cells detected by SA-β-Gal staining. β-gal staining area analysis was shown. Scale bar, 50 μm. **D** Immunofluorescence analysis of γ-H2AX expression in AML12 cells. Fluorescence intensity analysis was shown. Scale bar, 50 μm. Western blot analysis to measure the senescence-associated genes P53, P21 and P16 in AML12 cells treated with d-gal (**E**) or H_2_O_2_ (**F**). RT‒qPCR analysis of mRNA levels of senescence-associated secretory phenotype genes IL-6, IL-1β, TNF-α and NF-κB1 in AML12 cells treated with d-gal (**G**) or H_2_O_2_ (**H**). Data are shown as means ± SEM. One-way ANOVA was used for comparison among multiple groups. ^*^P < 0.05, ^**^P < 0.01, ^***^P < 0.001. All experiments were repeated three times independently

During RNA-Seq analysis, we found significantly elevated expression of CEBPB in *IGF2*^*f/f*^*Cre* + d-gal mice compared to *IGF2*^*f/f*^ + d-gal mice (Fig. 3A). As CEBPB plays an important role in senescence and SASPs, we hypothesized that CEBPB appears to be a key factor in regulating senescence and SASPs caused by IGF2 deficiency. In vivo experiments revealed that d-gal-treated mice increased CEBPB protein levels, and IGF2 knockout further elevated CEBPB (Fig. 6A). Similar results were observed during in vitro experiments; IGF2 knockdown increased the protein level of CEBPB in d-gal or H_2_O_2_-treated AML12 cells (Fig. 6B). To further ascertain the role of CEBPB in senescence caused by IGF2 deficiency, AML12 cells were transfected with sh-NC + si-NC, sh-NC + si-CEBPB, sh-IGF2 + si-NC and sh-IGF2 + si-CEBPB. Western blotting and RT‒qPCR revealed successful inhibition of CEBPB in AML12 cells (Additional file 4: Figure S3A, B).

We investigated whether CEBPB silencing would alleviate the pro-aging effect of IGF2 knockdown. IGF2 knockdown upregulated SA-β-gal activity in AML12 cells treated with d-gal or H_2_O_2_, while CEBPB silencing rescued this effect (Fig. 6C). Immunofluorescence staining showed that IGF2 knockdown increased DNA damage marker γ-H2AX expression in AML12 cells treated with d-gal or H_2_O_2_, which was reversed by CEBPB silencing (Fig. 6D). Consistently, IGF2 knockdown upregulated senescence genes P53, P21 and P16 in AML12 cells treated with d-gal, while CEBPB silencing partially rescued this effect by downregulating P53 and P21 (Fig. 6E). IGF2 knockdown upregulated P53 and P16 in AML12 cells treated with H_2_O_2,_ while CEBPB silencing rescued this effect (Fig. 6F). Furthermore, IGF2 knockdown restricted cell proliferation in AML12 cells treated with d-gal or H_2_O_2_, while CEBPB silencing prevented this effect (Additional file 4: Figure S3C, D). Meanwhile, IGF2 knockdown had a higher proportion of cells in the G1 phase in AML12 cells treated with d-gal, and CEBPB silencing rescued this effect (Additional file 4: Figure S3E). However, CEBPB silencing was not observed to reverse this effect in AML12 cells treated with H_2_O_2_ (Additional file 4: Figure S3F). Finally, IGF2 knockdown upregulated IL-6, TNF-α, and NF-κB1 in AML12 cells treated with d-gal or H_2_O_2_, while CEBPB silencing eliminated this effect (Fig. 6G, H).

These findings demonstrate that CEBPB silencing improves senescence phenotypes and SASPs caused by IGF2 knockdown in AML12 treated with d-gal or H_2_O_2_.

## Discussion

**Fig. 7:**
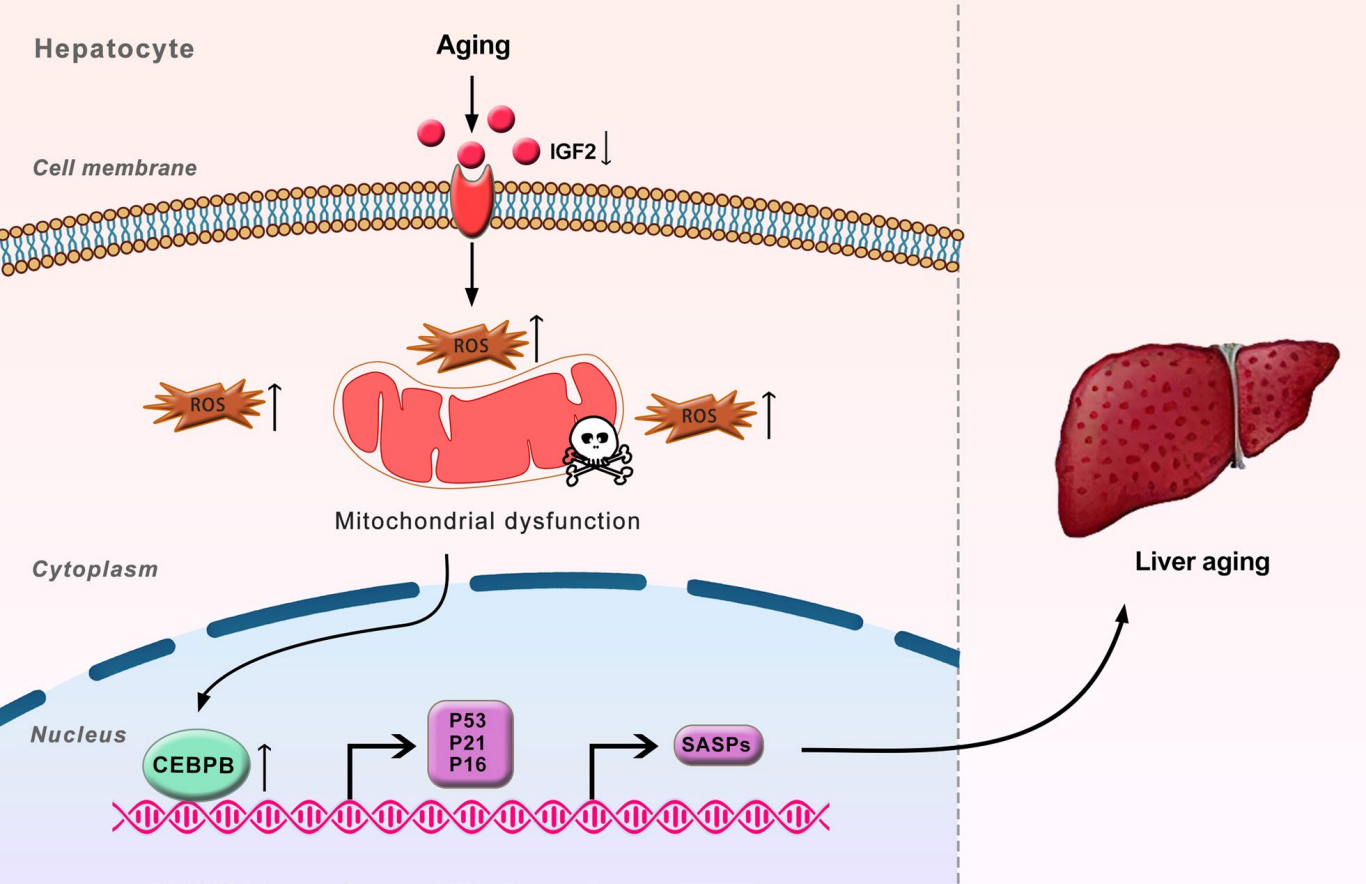
IGF2 deficiency promotes liver aging through mitochondrial dysfunction and upregulated CEBPB signaling. Schematic model: **A** during natural aging, the protein level of IGF2 is significantly decreased in the aged livers of old mice. **B** IGF2 deficiency in the liver leads to mitochondrial dysfunction, which leads to oxidative stress and cellular damage. Subsequently, oxidative stress and cellular damage are likely to activate ERK1/2 pathway and further upregulate CEBPB signaling, leading to senescence phenotypes and senescence-associated secretory phenotypes

It is widely understood that aging is the predominant risk factor for developing CLD (Papatheodoridi et al. 2020), implying that the underlying mechanisms of CLD involve cellular senescence and the aging microenvironment. Here, we showed that the livers of aged mice exhibited an aging microenvironment. Additionally, the expression of P21 and IL-1β were found to be upregulated even in middle-aged mice, indicating that liver aging processes can initiate in middle age. However, the levels of these markers did not exhibit a further increase in old mice compared to middle-aged mice. This observation might be attributed to the complex microenvironment of aging liver, which combines elements of both an aging microenvironment and a pro-tumor microenvironment. We also provide multiple lines of evidence showing the importance of IGF2 in liver aging (a graphic summary is shown in Fig. 7). Firstly, IGF2 was significantly downregulated in the liver of aged mice. Secondly, IGF2 suppression induced senescence phenotypes and SASPs through mitochondrial dysfunction and the upregulation of CEBPB signaling, both in vitro and in vivo aging models. To our knowledge, this study is the first to illustrate the effect of IGF2 deficiency on liver aging.

High doses of d-gal might induce senescence by the formation of advanced glycation end products, H_2_O_2_ and galactitol which leads to redox imbalance, ROS formation, and osmotic stress (Azman et al. 2021). H_2_O_2_, which can elevate intracellular ROS levels, has been employed extensively with various cell types to induce senescence, including hepatocytes (Aravinthan et al. 2014). In the current study, d-gal or H_2_O_2_ was used to induce senescence in AML12 cells in vitro to provide more evidence in our study. And we found that IGF2 knockdown in AML12 cells promoted senescence phenotypes and SASPs in these two senescent models, but the results in these two senescent models are not completely consistent. Differences in the mechanisms leading to senescence between d-gal and H_2_O_2_ may account for the discrepancy in the results. Consistently, IGF2 knockout accelerated d-gal-induced liver aging, which can increase the risk of aging-related CLD. Similar to our results, it has been reported that lower levels of IGF2 in plasma are associated with more severe liver fibrosis in patients with non-alcoholic fatty liver disease (Ajmera et al. 2017). Moreover, in the mouse model of chronic liver injury caused by tyrosinemia or long-term treatments of CCl4, IGF2 can significantly promote hepatocyte proliferation and tissue repair in the condition of chronic liver injury (Liu et al. 2017). However, in the mouse model of HCC, IGF2 was overexpressed in HCC mice, and deletion of IGF2 prevented DNA damage and HCC development (Kumar et al. 2022). The complicated role of IGF2 in CLD may be attributed to the hyperbolic effects of IGF2. A low dose of IGF2 (≤ 50 ng per mouse) alleviated colitis and inhibited inflammation, while high doses of IGF2 (1000 ng per mouse) failed to ameliorate colitis and instead exacerbated its progression and promoted inflammation (Wang et al. 2020).

One of the main characteristics of senescence is mitochondrial dysfunction, which is also the important trigger of aging (Martini and Passos 2023). In the current study, IGF2 suppression significantly disrupted mitochondrial functions, which included impaired OXPHOS function, decreased MMP, increased both intracellular and mitochondrial ROS levels, and disrupted the mitochondrial membrane structure. These alterations are closely interconnected with senescence phenotypes and play a crucial role in regulating the SASPs. Consistent with our findings, it has been reported that IGF2 deficiency led to mitochondrial dysfunction in the liver and skeletal muscle (Gui et al. 2021; Zhu et al. 2021). In addition, decreased NAD^+^/NADH ratio is reportedly a vital trigger of mitochondrial dysfunction-associated senescence (Wiley et al. 2016). Yang et al. documented that NAD^+^ levels gradually decline during ovarian aging, while NR supplementation, an NAD^+^ precursor, could improve mitochondrial functions and reverse ovarian aging (Yang et al. 2020). In our present study, we revealed that IGF2 knockdown reduced the NAD^+^/NADH ratio. To further clarify whether pro-aging effect of IGF2 deficiency is related to lower NAD^+^/NADH ratios, we performed NAD^+^ replenishment via supplementing with NR to increase NAD^+^ content in AML12 cells treated with d-gal or H_2_O_2_. We found that NR supplementation reversed the pro-aging effect of IGF2 deficiency to a certain extent, implying that IGF2 deficiency promotes senescence related to mitochondrial dysfunction.

IGF2 performs its functions by binding with three receptors, namely the insulin-like growth factor 2 receptor (IGF2R), insulin-like growth factor 1 receptor (IGF1R), and insulin receptor (IR; IGF-2 binds mostly with its IR-A isoform) (Selenou et al. 2020). The binding of IGF2 to IGF1R or IR leads to the activation of the mitogen-activated protein kinase (MAPK) and PI3-kinase/Akt signaling pathways, mainly regulating proliferation, differentiation, or both (Chao and D'Amore 2008; Pollak 2008). IGF2R, a mannose-6-phosphate cation-dependent receptor with high affinity with IGF2 and almost no binding to IGF1, has long been thought to be a scavenger of IGF2 through lysosomal degradation. However, recent studies reported that IGF2R interacting with IGF2 could trigger signaling pathways (Chen et al. 2011; Garcia-Huerta et al. 2020). Up to now, the molecular mechanism for IGF2 deficiency-induced mitochondrial dysfunction remains poorly understood. It has been reported that IGF2 therapy at a low dose could revert liver mitochondrial dysfunction in aging rats, and considered this effect similar to IGF1 (Garcia-Fernandez et al. 2011). However, growing evidence suggests that IGF2R is involved in IGF2-regulated mitochondrial function (Martin-Montanez et al. 2014, 2017). A recent study have suggested that low doses of IGF2 binding to IGF2R could promote mitochondrial OXPHOS via proton rechanneling (Wang et al. 2020). In the current study, it was observed that the phosphorylation of AKT, ERK1/2, which are known to be activated by the binding of IGF2 to IGF1R, was not inhibited with IGF2 knockout. Therefore, while the possibility of the IGF1R receptor being involved in IGF2-regulated mitochondrial function cannot be entirely ruled out, the contribution of this signaling pathway appears to be limited in this context.

CCAAT/enhancer-binding protein beta (CEBPB) is a member of the CCAAT/enhancer-binding protein family that can be activated by inflammatory and stress response (Tsukada et al. 2011; Krieken et al. 2015). An increasing body of evidence suggests that CEBPB plays an important role in oncogene-induced senescence and SASPs (Salotti and Johnson 2019). Our work showed that the expression of CEBPB was significantly upregulated by IGF2 suppression, indicating the critical role of CEBPB in pro-aging of IGF2 deficiency. Furthermore, CEBPB inhibition reversed senescence phenotypes and reduced SASPs induced by IGF2 deficiency. We provide compelling evidence that IGF2 deficiency promotes senescence and SASPs by upregulating CEBPB signaling. In line with our study, previous research showed that CEBPB overexpression induced senescence and significantly increased SASPs in LNCaP cells, while the suppression of CEBPB had the opposite effect (Barakat et al. 2015). Besides, another group demonstrated that CEBPB could efficiently bind to the P53 promoter and induce the expression of P53 in response to mitogen stimulation (Boggs and Reisman 2007), implying that CEBPB can depress cell growth and promote senescence via upregulating P53 expression. Moreover, CEBPB is a target of Ras signaling and is mainly activated by the ERK1/2 (Salotti and Johnson 2019; Lee et al. 2010). Our study confirmed that the absence of IGF2 leads to the activation of the ERK1/2 pathway, which can be triggered by DNA damage and oxidative stress, subsequently promoting both DNA damage and oxidative stress (Anerillas et al. 2020). This finding suggests that the increased CEBPB signaling may be associated with the activation of the ERK1/2 pathway, which might be activiated by mitochondrial dysfunction.

## Conclusions

In summary, our study underscores the significant contribution of IGF2 deficiency in inducing a senescence phenotypes and SASPs in the liver of d-gal-induced aging mice and senescent AML12 cells. Mechanistically, the detrimental effects of IGF2 deficiency on aging processes are mediated through mitochondrial dysfunction and the upregulation of CEBPB signaling. These findings offer valuable insights into the potential mechanisms that connect IGF2 deficiency with liver aging. Consequently, IGF2 may emerge as a promising target for interventions aimed at ameliorating liver aging under specific circumstances.

### Supplementary Information

**Additional file 1: Table S1.** The sequences of shRNA and siRNA used in the study. **Table S2.** The primer sequences used in the study.

**Additional file 2: Figure S1.** Effects of IGF2 knockout on metabolism. (**A**) Targeting strategy of generating *IGF2* floxed (*IGF2*^*f/f*^) mice by CRISPR-Cas9 technology. (**B**) PCR analysis for mice genotypes. (**C**) Genetic analysis for mice genotypes. Body weight change (**D**), body weight gain (**E**) and ratios of liver/body weight (**F**) in *IGF2*^*f/f*^ and *IGF2*^*f/f*^*Cre* mice of saline control group and d-gal model group. Blood glucose levels (**G**) and AUC (**H**) during intraperitoneal glucose tolerance test in *IGF2*^*f/f*^ and *IGF2*^*f/f*^*Cre* mice of saline control group and d-gal model group. Blood glucose levels (**I**) and AUC (**J**) during insulin tolerance test in *IGF2*^*f/f*^ and *IGF2*^*f/f*^*Cre* mice of saline control group and d-gal model group. Fasting glucose (**K**) and fasting insulin (**L**) levels in *IGF2*^*f/f*^ and *IGF2*^*f/f*^*Cre* mice of saline control group and d-gal model group. n = 5–7 per group. All values are shown as means ± SEM. Dots represent individual level data. One-way ANOVA was used for comparison among multiple groups. ^*^ *P* < 0.05, ^**^P < 0.01, ^***^P < 0.001.

**Additional file 3: Figure S2.** Effects of IGF2 knockout on transcriptomic changes. KEGG pathway enrichment analysis was conducted on the upregulated DEGs (**A**) and downregulated DEGs (**B**) in the liver tissues of *IGF2*^*f/f*^ + d-gal and *IGF2*^*f/f*^*Cre* + d-gal mice (n = 3 per group).

**Additional file 4: Figure S3.** Inhibition of CEBPB enhances cell viability in AML12 cells after IGF2 knockdown. Western blotting analysis (**A**) and RT‒qPCR analysis (**B**) for the expression of CEBPB protein and mRNA in AML12 cells transfected with si-CEBPB or si-NC. (C-F) AML12 cells were transfected with sh-NC + si-NC, sh-NC + si-CEBPB, sh-IGF2 + si-NC and sh-IGF2 + si-CEBPB, and treated with d-gal or H_2_O_2_. The cell viability tested by CCK-8 assay in AML12 cells treated with d-gal (**C**) or H_2_O_2_ (**D**). The cell cycle distribution analyzed by flow cytometry in AML12 cells treated with d-gal (**E**) or H_2_O_2_ (**F**). Data are shown as means ± SEM. Student t tests was used for comparison between two groups. One-way ANOVA was used for comparison among multiple groups. ^*^ *P* < 0.05, ^**^P < 0.01, ^***^P < 0.001. All experiments were repeated three times independently.

## Data Availability

The datasets used and/or analysed during the current study are available from the corresponding author on reasonable request.

## Abbreviations

Alb-cre: Albumin-cre
ALT: Alanine aminotransferase
AST: Aspartate aminotransferase
CEBPB: CCAAT/enhancer-binding protein beta
CLD: Chronic liver disease
CCK-8: Cell counting kit-8
D-Gal: D-Galactose
DMEM: Dulbecco’s modified Eagle’s medium
DAPI: 4′,6-Diamidino-2-phenylindole
DCFH-DA: 2′,7′-Dichlorodihydrofluorescein diacetate
DEGs: Differentially expressed genes
ELISA: Enzyme-linked immunosorbent assay
FCCP: Carbonyl cyanide 4(trifluoromethoxy)phenylhydrazone
GO: Gene Ontology
GSEA: Gene set enrichment analysis
HCC: Hepatocellular carcinoma
H_2_O_2_: Hydrogen peroxide
H&E: Hematoxylin and eosin
IGF2: Insulin-like growth factor 2
IGF1: Insulin-like growth factor 1
IPGTT: Intraperitoneal glucose tolerance testing
ITT: Insulin tolerance test
IGF2R: Insulin-like growth factor 2 receptor
IGF1R: Insulin-like growth factor 1 receptor
IR: Insulin receptor
KEGG: Kyoto Encyclopedia of Genes and Genomes
Log2FC: Log2 fold change
MOI: Multiplicity of infection
MMP: Mitochondrial membrane potential
MAPK: Mitogen-activated protein kinase
NAD: Nicotinamide adenine dinucleotide
NR: Nicotinamide riboside
OXPHOS: Oxidative phosphorylation
OCR: Oxygen consumption rate
RT‒qPCR: Real-time quantitative PCR
Rot/AA: Rotenone/antimycin A
ROS: Reactive oxygen species
SASPs: Senescence-associated secretory phenotypes
shRNA: Short hairpin RNA
siRNAs: Small interfering RNAs
SDS-PAGE: Sodium dodecylsulfate-polyacrylamide gel electrophoresis
SA-β-gal: Senescence-associated-β-galactosidase
MAPK: Mitogen-activated protein kinase

## References

[CR1] Adamek A, Kasprzak A (2018). Insulin-like growth factor (IGF) system in liver diseases. Int J Mol Sci.

[CR2] Ajmera V, Perito ER, Bass NM, Terrault NA, Yates KP, Gill R (2017). Novel plasma biomarkers associated with liver disease severity in adults with nonalcoholic fatty liver disease. Hepatology.

[CR3] Anerillas C, Abdelmohsen K, Gorospe M (2020). Regulation of senescence traits by MAPKs. Geroscience.

[CR4] Aravinthan A, Shannon N, Heaney J, Hoare M, Marshall A, Alexander GJ (2014). The senescent hepatocyte gene signature in chronic liver disease. Exp Gerontol.

[CR5] Azman KF, Safdar A, Zakaria R (2021). D-galactose-induced liver aging model: Its underlying mechanisms and potential therapeutic interventions. Exp Gerontol.

[CR6] Barakat DJ, Zhang J, Barberi T, Denmeade SR, Friedman AD, Paz-Priel I (2015). CCAAT/enhancer binding protein beta controls androgen-deprivation-induced senescence in prostate cancer cells. Oncogene.

[CR7] Beletskiy A, Chesnokova E, Bal N (2021). Insulin-like growth factor 2 as a possible neuroprotective agent and memory enhancer-its comparative expression, processing and signaling in mammalian CNS. Int J Mol Sci.

[CR8] Bergman D, Halje M, Nordin M, Engstrom W (2013). Insulin-like growth factor 2 in development and disease: a mini-review. Gerontology.

[CR9] Boggs K, Reisman D (2007). C/EBPbeta participates in regulating transcription of the p53 gene in response to mitogen stimulation. J Biol Chem.

[CR10] Cai Y, Song W, Li J, Jing Y, Liang C, Zhang L (2022). The landscape of aging. Sci China Life Sci.

[CR11] Calcinotto A, Kohli J, Zagato E, Pellegrini L, Demaria M, Alimonti A (2019). Cellular senescence: aging, cancer, and injury. Physiol Rev.

[CR12] Castilla-Cortazar I, Garcia-Fernandez M, Delgado G, Puche JE, Sierra I, Barhoum R (2011). Hepatoprotection and neuroprotection induced by low doses of IGF-II in aging rats. J Transl Med.

[CR13] Chao W, D'Amore PA (2008). IGF2: epigenetic regulation and role in development and disease. Cytokine Growth Factor Rev.

[CR14] Chen DY, Stern SA, Garcia-Osta A, Saunier-Rebori B, Pollonini G, Bambah-Mukku D (2011). A critical role for IGF-II in memory consolidation and enhancement. Nature.

[CR15] Garcia-Fernandez M, Sierra I, Puche JE, Guerra L, Castilla-Cortazar I (2011). Liver mitochondrial dysfunction is reverted by insulin-like growth factor II (IGF-II) in aging rats. J Transl Med.

[CR16] Garcia-Huerta P, Troncoso-Escudero P, Wu D, Thiruvalluvan A, Cisternas-Olmedo M, Henriquez DR (2020). Insulin-like growth factor 2 (IGF2) protects against Huntington's disease through the extracellular disposal of protein aggregates. Acta Neuropathol.

[CR17] Gui W, Zhu Y, Sun S, Zhu W, Tan B, Zhao H (2021). Knockdown of insulin-like growth factor 2 gene disrupts mitochondrial functions in the liver. J Mol Cell Biol.

[CR18] Hunt NJ, Kang SWS, Lockwood GP, Le Couteur DG, Cogger VC (2019). Hallmarks of aging in the liver. Comput Struct Biotechnol J.

[CR19] Kumar D, Das M, Oberg A, Sahoo D, Wu P, Sauceda C (2022). Hepatocyte deletion of IGF2 prevents DNA damage and tumor formation in hepatocellular carcinoma. Adv Sci (weinh).

[CR20] Lee BY, Han JA, Im JS, Morrone A, Johung K, Goodwin EC (2006). Senescence-associated beta-galactosidase is lysosomal beta-galactosidase. Aging Cell.

[CR21] Lee S, Shuman JD, Guszczynski T, Sakchaisri K, Sebastian T, Copeland TD (2010). RSK-mediated phosphorylation in the C/EBPbeta leucine zipper regulates DNA binding, dimerization, and growth arrest activity. Mol Cell Biol.

[CR22] Liu J, Hu X, Chen J, Li X, Wang L, Wang B (2017). Pericentral hepatocytes produce insulin-like growth factor-2 to promote liver regeneration during selected injuries in mice. Hepatology.

[CR23] Maeso-Diaz R, Gracia-Sancho J (2020). Aging and Chronic liver disease. Semin Liver Dis.

[CR24] Martini H, Passos JF (2023). Cellular senescence: all roads lead to mitochondria. FEBS J.

[CR25] Martin-Montanez E, Pavia J, Santin LJ, Boraldi F, Estivill-Torrus G, Aguirre JA (2014). Involvement of IGF-II receptors in the antioxidant and neuroprotective effects of IGF-II on adult cortical neuronal cultures. Biochim Biophys Acta.

[CR26] Martin-Montanez E, Millon C, Boraldi F, Garcia-Guirado F, Pedraza C, Lara E (2017). IGF-II promotes neuroprotection and neuroplasticity recovery in a long-lasting model of oxidative damage induced by glucocorticoids. Redox Biol.

[CR27] Martin-Montanez E, Valverde N, Ladron Guevara-Miranda D, Lara E, Romero-Zerbo YS, Millon C (2021). Insulin-like growth factor II prevents oxidative and neuronal damage in cellular and mice models of Parkinson's disease. Redox Biol.

[CR28] Miwa S, Kashyap S, Chini E, von Zglinicki T (2022). Mitochondrial dysfunction in cell senescence and aging. J Clin Invest.

[CR29] Muhammad T, Wan Y, Sha Q, Wang J, Huang T, Cao Y (2020). IGF2 improves the developmental competency and meiotic structure of oocytes from aged mice. Aging (albany NY).

[CR30] Papatheodoridi AM, Chrysavgis L, Koutsilieris M, Chatzigeorgiou A (2020). The role of senescence in the development of nonalcoholic fatty liver disease and progression to nonalcoholic steatohepatitis. Hepatology.

[CR31] Pollak M (2008). Insulin and insulin-like growth factor signalling in neoplasia. Nat Rev Cancer.

[CR32] Rotwein P (1991). Structure, evolution, expression and regulation of insulin-like growth factors I and II. Growth Factors.

[CR33] Rui L (2014). Energy metabolism in the liver. Compr Physiol.

[CR34] Salotti J, Johnson PF (2019). Regulation of senescence and the SASP by the transcription factor C/EBPbeta. Exp Gerontol.

[CR35] Schmucker DL (2005). Age-related changes in liver structure and function: implications for disease ?. Exp Gerontol.

[CR36] Selenou C, Brioude F, Giabicani E, Sobrier ML, Netchine I (2022). IGF2: development, genetic and epigenetic abnormalities. Cells.

[CR37] Steinmetz AB, Johnson SA, Iannitelli DE, Pollonini G, Alberini CM (2016). Insulin-like growth factor 2 rescues aging-related memory loss in rats. Neurobiol Aging.

[CR38] Tang H, Yao F, Yin M, Liao Y, Li K, Li L (2022). Anti-senescent effects of long non-coding RNA H19 on human dermal fibroblast cells through impairing microRNA-296-5p-dependent inhibition of IGF2. Cell Signal.

[CR39] Tsukada J, Yoshida Y, Kominato Y, Auron PE (2011). The CCAAT/enhancer (C/EBP) family of basic-leucine zipper (bZIP) transcription factors is a multifaceted highly-regulated system for gene regulation. Cytokine.

[CR40] van der Krieken SE, Popeijus HE, Mensink RP, Plat J (2015). CCAAT/enhancer binding protein beta in relation to ER stress, inflammation, and metabolic disturbances. Biomed Res Int.

[CR41] Vu TH, Hoffman AR (1994). Promoter-specific imprinting of the human insulin-like growth factor-II gene. Nature.

[CR42] Wang X, Lin L, Lan B, Wang Y, Du L, Chen X (2020). IGF2R-initiated proton rechanneling dictates an anti-inflammatory property in macrophages. Sci Adv.

[CR43] Wiley CD, Velarde MC, Lecot P, Liu S, Sarnoski EA, Freund A (2016). Mitochondrial dysfunction induces senescence with a distinct secretory phenotype. Cell Metab.

[CR44] Yang Q, Cong L, Wang Y, Luo X, Li H, Wang H (2020). Increasing ovarian NAD(+) levels improve mitochondrial functions and reverse ovarian aging. Free Radic Biol Med.

[CR45] Zhu Y, Gui W, Tan B, Du Y, Zhou J, Wu F (2021). IGF2 deficiency causes mitochondrial defects in skeletal muscle. Clin Sci (lond).

