# Supplementary material for: IGF2 deficiency promotes liver aging through mitochondrial dysfunction and upregulated CEBPB signaling in d-galactose-induced aging mice
Source: Mol Med. 2023 Nov 28;29:161. doi: 10.1186/s10020-023-00752-0 (PMC10685569; doi:10.1186/s10020-023-00752-0)
Supplement: Supplementary file 1 — Additional file 1: Table S1. The sequences of shRNA and siRNA used in the study. Table S2. The primer sequences used in the study. [file 10020_2023_752_MOESM1_ESM.docx]

**Table S1**

The sequences of shRNA and siRNA used in the study.

|  | **Sequences** |
| --- | --- |
| sh-NC | TTCTCCGAACGTGTCACGT |
| sh-IGF2 | ATCGTTGAGGAGTGCTGTT |
| si-NC | GAAUUGCUCUCGGACAAUUCG |
| si-CEBPB | CACCCUGCGGAACUUGUUCAA |

**Table S2**

The primer sequences used in the study.

| **Gene** | **Forward (5’-3’)** | **Reverse (5’-3’)** |
| --- | --- | --- |
| GAPDH | ATGAC ATCAA GAAGG TGGTG AAGC | GAAGA GTGGG AGTTG CTGTT GAAG |
| IGF2 | TGGTGCTTCTCATCTCTTTGG | GAACAGACAAACTGAAGCGTG |
| P53 | CCCCTGTCATCTTTTGTCCCT | AGCTGGCAGAATAGCTTATTGAG |
| P21 | CCTGGTGATGTCCGACCTG | CCATGAGCGCATCGCAATC |
| P16 | GAACTCTTTCGGTCGTACCC | CGAATCTGCACCGTAGTTGA |
| IL-6 | GAGGA TACCA CTCCC AACAG ACC | AAGTG CATCA TCGTT GTTCA TACA |
| IL-1β | ACTCC TTAGT CCTCG GCCA | CCATC AGAGG CAAGG AGGAA |
| TNF-α | TGATC CGCGA CGTGG AA | ACCGC CTGGA GTTCT GGAA |
| NF-κB1 | GTCTCAAACCAAACAGCCTCAC | CAGTGTCTTCCTCGACATGGAT |
| CEBPB | GTTTCGGGACTTGATGCAAT | CCCCGCAGGAACATCTTTA |
