# Supplementary figures and images for: IGF2 deficiency promotes liver aging through mitochondrial dysfunction and upregulated CEBPB signaling in d-galactose-induced aging mice
Source: Mol Med. 2023 Nov 28;29:161. doi: 10.1186/s10020-023-00752-0 (PMC10685569; doi:10.1186/s10020-023-00752-0)

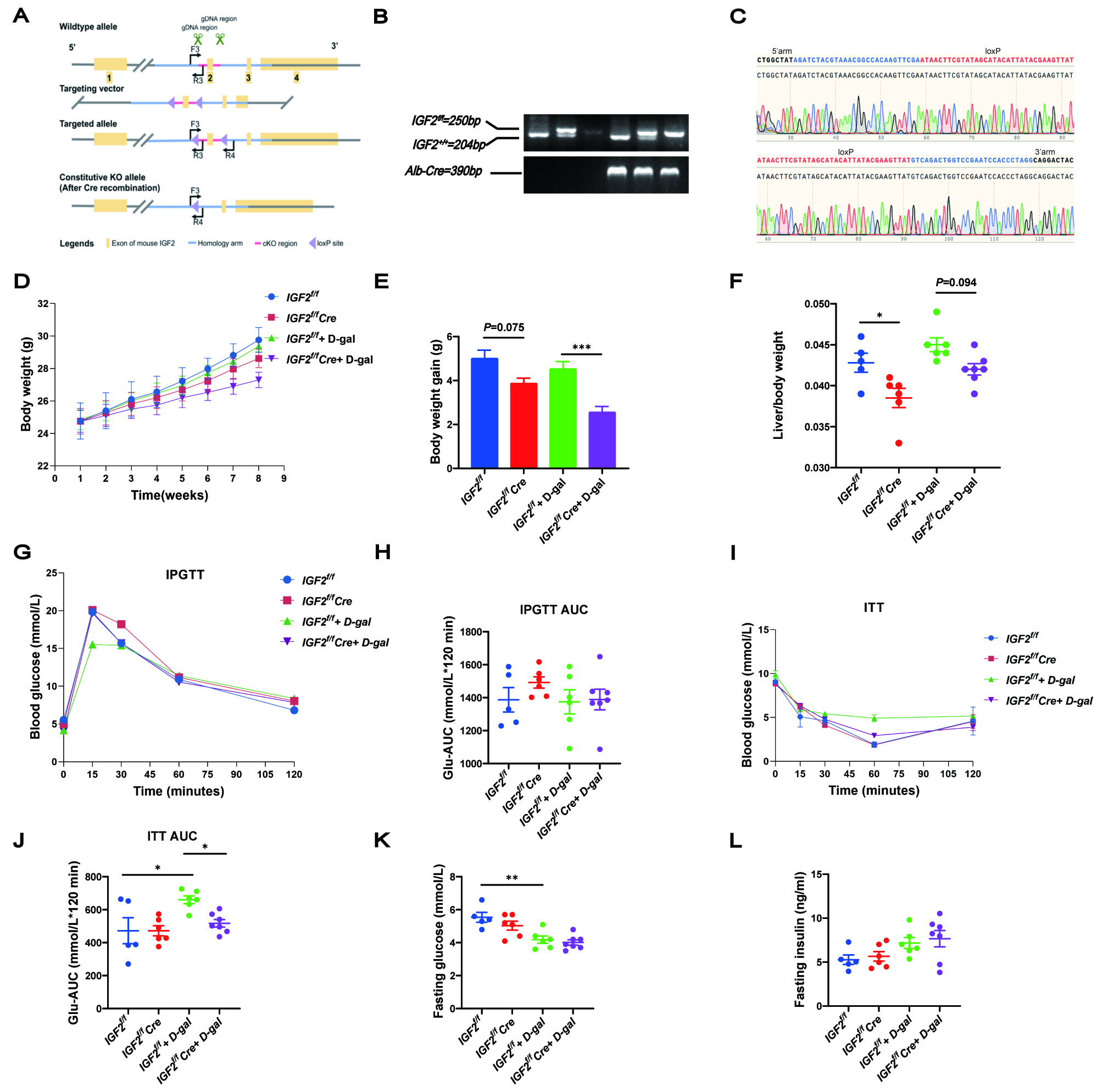

Supplement: Supplementary file 2 — Additional file 2: Figure S1. Effects of IGF2 knockout on metabolism. (A) Targeting strategy of generating IGF2 floxed (IGF2f/f) mice by CRISPR-Cas9 technology. (B) PCR analysis for mice genotypes. (C) Genetic analysis for mice genotypes. Body weight change (D), body weight gain (E) and ratios of liver/body weight (F) in IGF2f/f and IGF2f/fCre mice of saline control group and d-gal model group. Blood glucose levels (G) and AUC (H) during intraperitoneal glucose tolerance test in IGF2f/f and IGF2f/fCre mice of saline control group and d-gal model group. Blood glucose levels (I) and AUC (J) during insulin tolerance test in IGF2f/f and IGF2f/fCre mice of saline control group and d-gal model group. Fasting glucose (K) and fasting insulin (L) levels in IGF2f/f and IGF2f/fCre mice of saline control group and d-gal model group. n = 5–7 per group. All values are shown as means ± SEM. Dots represent individual level data. One-way ANOVA was used for comparison among multiple groups. * P < 0.05, **P < 0.01, ***P < 0.001. [file 10020_2023_752_MOESM2_ESM.tif]

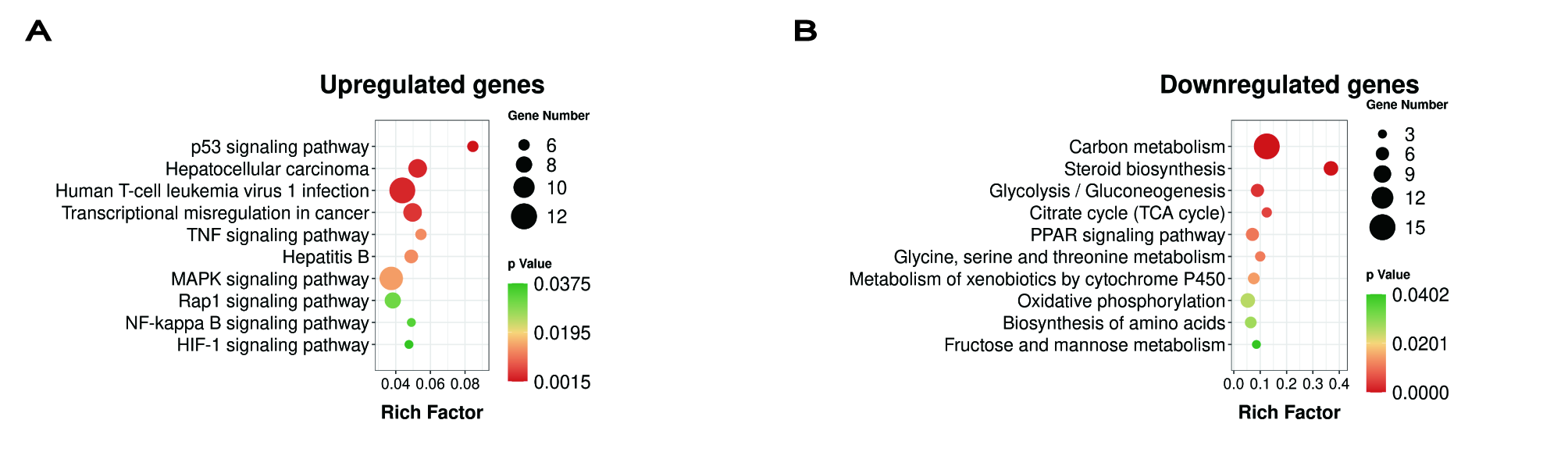

Supplement: Supplementary file 3 — Additional file 3: Figure S2. Effects of IGF2 knockout on transcriptomic changes. KEGG pathway enrichment analysis was conducted on the upregulated DEGs (A) and downregulated DEGs (B) in the liver tissues of IGF2f/f + d-gal and IGF2f/fCre + d-gal mice (n = 3 per group). [file 10020_2023_752_MOESM3_ESM.tif]

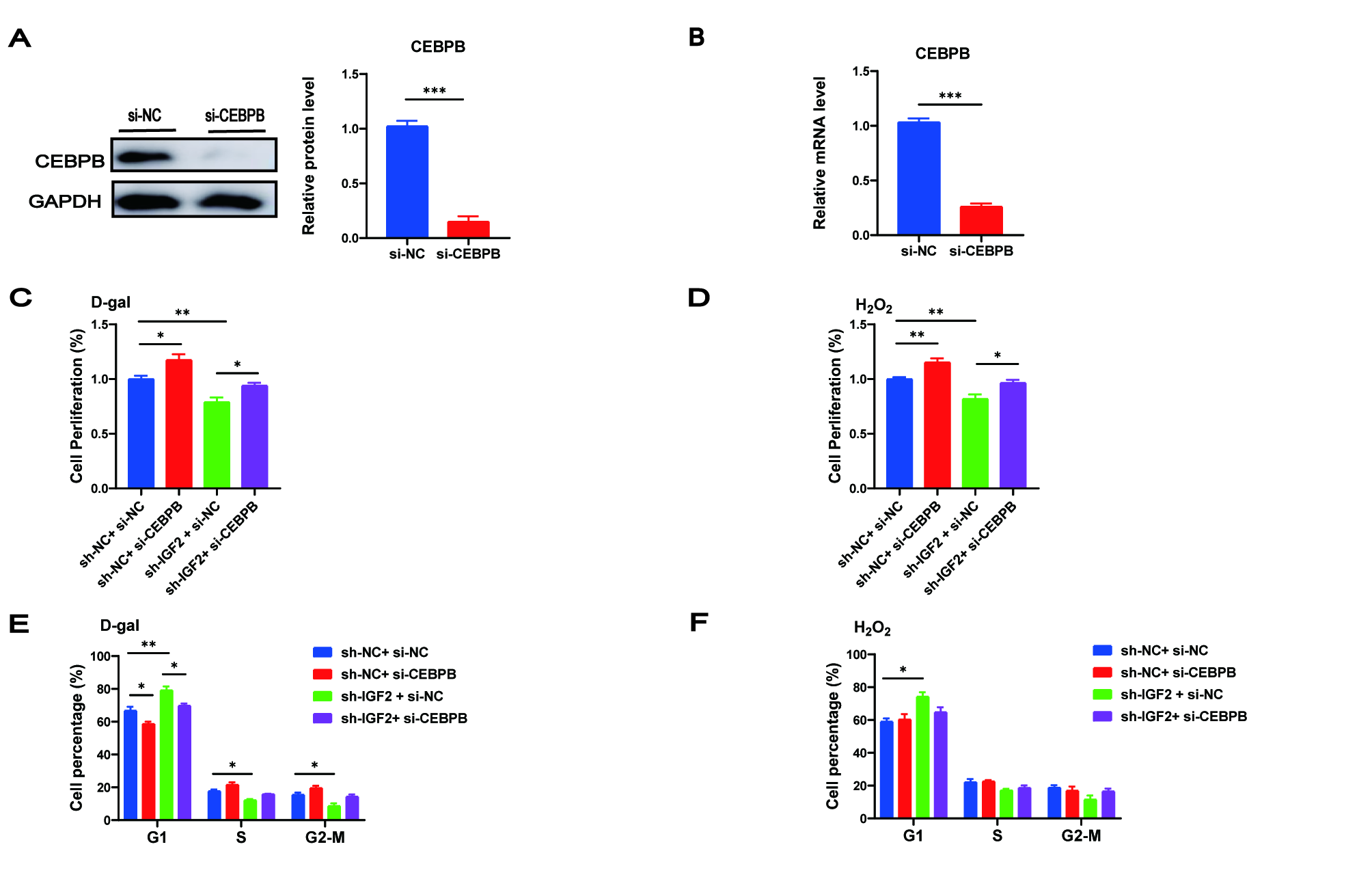

Supplement: Supplementary file 4 — Additional file 4: Figure S3. Inhibition of CEBPB enhances cell viability in AML12 cells after IGF2 knockdown. Western blotting analysis (A) and RT‒qPCR analysis (B) for the expression of CEBPB protein and mRNA in AML12 cells transfected with si-CEBPB or si-NC. (C-F) AML12 cells were transfected with sh-NC + si-NC, sh-NC + si-CEBPB, sh-IGF2 + si-NC and sh-IGF2 + si-CEBPB, and treated with d-gal or H2O2. The cell viability tested by CCK-8 assay in AML12 cells treated with d-gal (C) or H2O2 (D). The cell cycle distribution analyzed by flow cytometry in AML12 cells treated with d-gal (E) or H2O2 (F). Data are shown as means ± SEM. Student t tests was used for comparison between two groups. One-way ANOVA was used for comparison among multiple groups. * P < 0.05, **P < 0.01, ***P < 0.001. All experiments were repeated three times independently. [file 10020_2023_752_MOESM4_ESM.tif]
